# Biosocial influences of gender identity and geography on mucosal microbial phenotypes

**DOI:** 10.1038/s43856-026-01780-7

**Published:** 2026-07-14

**Authors:** Vanessa E. Van Doren, S. Abigail Smith, Cassie Grimsley Ackerley, Jadah Keith, Robert A. Arthur, Henry Claussen, Phillip Murray, Vin Tangpricha, Yi-Juan Hu, Chang Su, Mengyu He, Colleen F. Kelley

**Affiliations:** 1 The Hope Clinic of the Emory Vaccine Center, Division of Infectious Diseases, Department of Medicine, Emory University School of Medicine, Decatur, GA USA; 2 Emory University School of Medicine, Atlanta, GA USA; 3 Emory Integrated Computational Core, Emory University, Woodruff Memorial Research Building, Atlanta, GA USA; 4 Division of Endocrinology, Metabolism, and Lipids, Department of Medicine, Emory University School of Medicine, Atlanta, GA USA; 5 Department of Biostatistics and Bioinformatics, Rollins School of Public Health, Emory University, Atlanta, GA USA; 6 Grady Health System, 80 Jesse Hill Jr Drive, Atlanta, GA USA

**Keywords:** Clinical microbiology, Microbiome, Endocrine system and metabolic diseases, Medical research

## Abstract

**Background:**

Transgender women (TGW) experience unique hormonal contexts and high HIV incidence, yet the mucosal microbiome among TGW remains understudied. Sex hormones and geography may shape microbial composition, but the relative contributions of gender identity, feminizing hormone therapy (FHT), and location are unclear.

**Methods:**

We conducted a multi-site study of TGW using FHT and cisgender men who have sex with men (MSM), all without HIV, in Atlanta, USA (*n* = 58; 25 TGW, 33 MSM) and Bangkok, Thailand (*n* = 147; 97 TGW, 50 MSM)(*n* = 205). We also conducted longitudinal sampling in 21 TGW pre/post FHT initiation. Rectal swabs were collected from all participants, with optional neovaginal sampling in TGW. Microbiota composition was analyzed using 16S rRNA sequencing, and associations with gender category, geography, and hormone concentrations were assessed using linear decomposition modeling (LDM) and BOUTH analysis.

**Results:**

Here we show that the rectal microbiota differ significantly by both gender category and geography. TGW exhibit enrichment of estrogen-metabolizing taxa across sites, while MSM show *Prevotellaceae* enrichment in Atlanta only. Alpha diversity varies by location but not gender category. Neovaginal microbiota differ markedly from rectal composition, showing enrichment of skin- and gut-associated taxa and anaerobic taxa associated with HIV seroconversion. No significant rectal microbiota shifts are observed after short-term FHT initiation, possibly reflecting subtherapeutic hormone exposure.

**Conclusions:**

These findings underscore the need to consider gender identity as a complex biosocial phenotype in HIV prevention and highlight the potential role of mucosal microbiota in shaping HIV vulnerability in TGW.

## Plain language summary

Transgender women are at elevated risk of acquiring HIV, but little is known about the bacteria that normally live in their bodies and how these might affect health. We studied whether gender identity, hormone use, and geographic location influence these bacteria. We collected rectal samples from transgender women and cisgender men in the United States and Thailand, along with neovaginal samples from some transgender women in Thailand. We also followed a small group of transgender women before and after starting hormone therapy. We found that bacterial communities differed by gender identity and location. Transgender women had more estrogen-linked bacteria, while location influenced overall diversity. Short-term hormone therapy had little effect. These results show that social and biological factors together shape the microbiome and may affect HIV risk.

## Abstract (web summary)

Van Doren et al. examine how gender identity and geography shape mucosal microbiomes in transgender women and cisgender men. Their findings show biosocial influences on microbial communities that may help explain differences in HIV susceptibility.

## Introduction

The intestinal microbiota is a critical determinant of mucosal immune function, epithelial barrier integrity, and metabolic signaling^1–3^. Rectal mucosal communities, in particular, influence susceptibility to enteric and sexually transmitted infections, including HIV, by modulating local inflammation and availability of viral target cells^4^. Among cisgender men who have sex with men (MSM), receptive anal intercourse (RAI) is associated with distinct rectal microbiota features, often characterized by *Prevotella* enrichment and reduced microbial diversity, that have been linked to mucosal immune activation and HIV acquisition^5,6^. However, little is known about the rectal microbiota in transgender women (TGW), a population with disproportionately high HIV incidence and frequent use of feminizing hormone therapy (FHT) that could influence microbiota composition.

Sex hormones are emerging as key regulators of gut microbial ecology, with growing evidence from experimental and human studies that both androgens and estrogens shape microbial community composition through multiple pathways, including modulation of bile acid metabolism, epithelial barrier function, and mucosal immune tone^7–10^. The gut microbiota can also metabolize and recycle both androgens and estrogens through microbial β-glucuronidase activity and enterohepatic recirculation^11,12^. These bidirectional interactions have been implicated in cardiometabolic, autoimmune, and oncologic outcomes that differ by sex, yet their relevance to mucosal sites implicated in HIV transmission or among people who use gender-affirming hormone therapy remains poorly defined. While systemic estrogen exposure and testosterone suppression through FHT profoundly alter circulating sex hormone concentrations, their impact on mucosal microbial composition in TGW remains understudied^13,14^. Geographic variation in diet, antibiotic exposure, sexual health practices, and access to gender-affirming care can also substantially shape mucosal microbial communities^15–17^, potentially obscuring or confounding hormone-related signals. *Prevotella* enrichment has been observed among MSM in both the United States and Europe^18–21^ but has not been assessed in TGW or across broader geographic contexts. Understanding whether gender category-associated microbial phenotypes persist across such contexts is essential for defining biologically conserved features of mucosal immunity relevant to HIV prevention.

To address these knowledge gaps, we conducted a multi-site study of individuals assigned male at birth: cisgender MSM and TGW using FHT who have sex with men in Atlanta, Georgia, USA and Bangkok, Thailand. Using cross-sectional and longitudinal study designs, we investigated how gender identity, geography, and FHT are associated with rectal mucosal microbiota composition using a recently developed, phylogeny-informed statistical framework that identifies key driver taxa that contribute to community differences and facilitates interpretation of complex microbiome signals. We further characterized neovaginal microbial communities in a subset of TGW. By integrating behavioral, hormonal, and microbiota data, this study aimed to delineate biological and contextual factors shaping mucosal microbial phenotypes that may underlie HIV vulnerability in gender and geographically diverse populations. We find that rectal microbiota composition differs significantly by both gender category and geography, with enrichment of taxa previously associated with estrogen metabolism among TGW, and *Prevotellaceae* enrichment limited to Atlanta MSM. Neovaginal communities harbor taxa previously linked to mucosal inflammation and HIV susceptibility, highlighting potential implications for HIV prevention research.

## Methods

### The clinical cohort

**Fig. 1:**
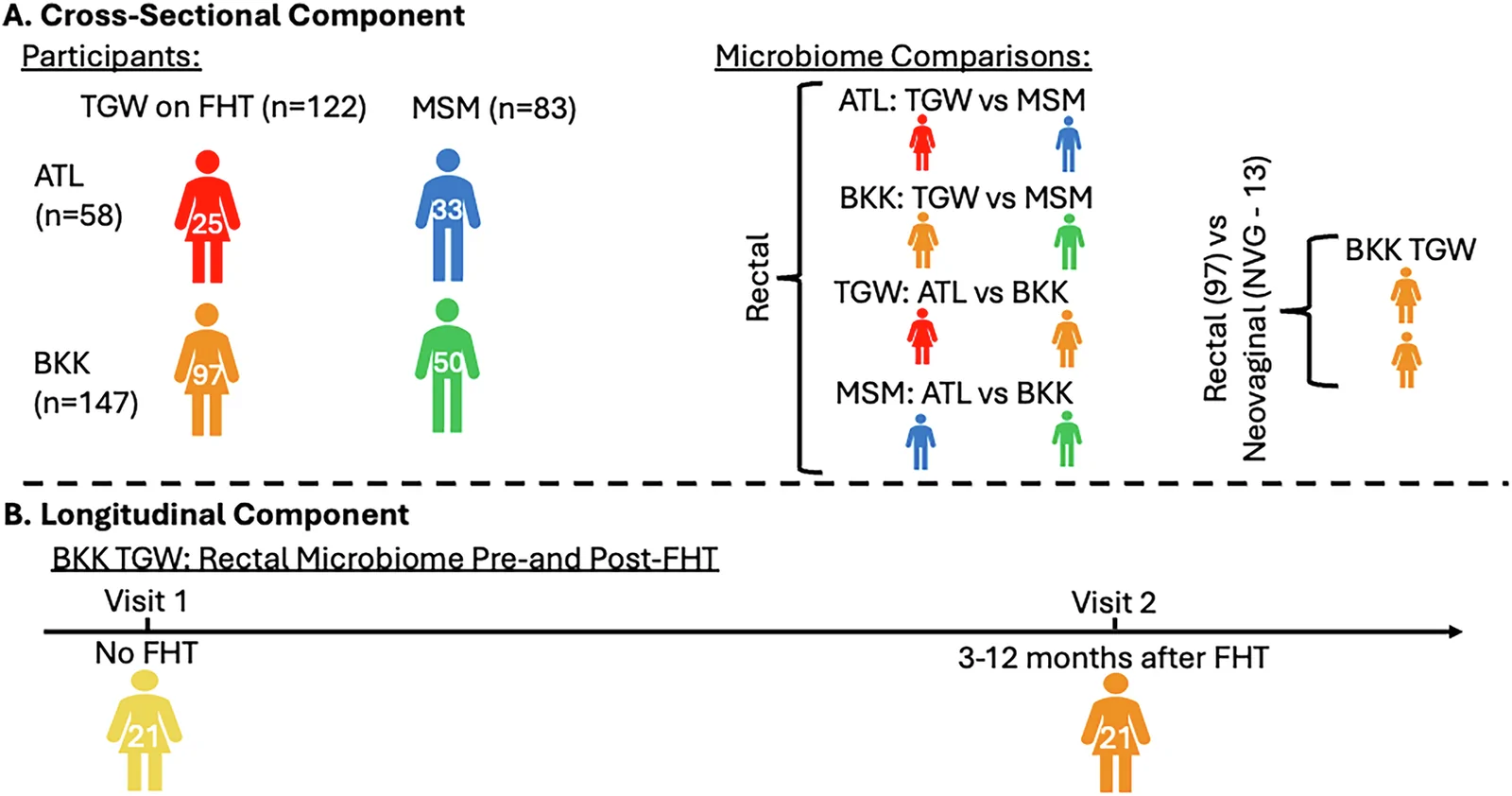
Study design. We compared the mucosal microbiota in key populations using two complementary observational study designs – cross-sectional and longitudinal. **A** For the cross-sectional component, we compared the rectal microbiota between transgender women (TGW) and cisgender men who have sex with men (MSM) in Atlanta (ATL); TGW and MSM in Bangkok (BKK); TGW in ATL versus BKK; and MSM in ATL versus BKK. We also compared the rectal and neovaginal microbiota in a subset of TGW in BKK. **B** For the longitudinal component, we compared the rectal microbiota before and 3–12 months after feminizing hormone therapy (FHT) initiation in 21 TGW from BKK.

MSM and TGW aged 18–59 years without HIV and in good health were recruited in Atlanta, Georgia, USA (ATL) and Bangkok, Thailand (BKK) between October 2020 and April 2024. Gender category (MSM vs TGW) was based on self-reported identity and used as a biosocial grouping variable reflecting hormonal, behavioral, and clinical differences. TGW participants were receiving FHT, representing a subset of transfeminine individuals. The study included cross-sectional and longitudinal components (Fig. 1). Approvals were obtained from the Emory University IRB (CR005-IRB00112414) and the Bamrasnaradura Infectious Diseases Institute IRB (Code P006h/63), and all participants provided informed consent. The parent study was registered at ClinicalTrials.gov (NCT04531943).

### Cross-sectional study

A total of 145 TGW on FHT (45 ATL; 100 BKK) and 94 MSM (44 ATL; 50 BKK) were enrolled. TGW participants had used FHT (oral, patch, topical, or injection estrogen in any dosage with or without anti-androgens and/or progestin) for at least six months and with stable dosing for at least three months prior to sampling. All participants reported lifetime RAI, which is known to influence the rectal microbiota^6,21–23^. In ATL, 13 TGW and 9 MSM did not complete the study; all BKK participants completed the study. Low-quality samples precluded analysis in 2 ATL TGW, 1 ATL MSM, and 3 BKK TGW. An additional 5 ATL TGW and 1 ATL MSM samples were excluded due to sequencing timing. Final analyses included 25 ATL TGW, 33 ATL MSM, 97 BKK TGW, and 50 BKK MSM.

Rectal mucosal tissue for immune phenotyping and HIV explant assays was collected as part of the parent study; these results are reported separately (Smith et al, manuscript under review). Use of synthetic estradiol formulations for FHT is common in Bangkok but uncommon in the United States^24^. Estradiol formulations were classified as 17β estradiol (detectable by assay: 17β-estradiol gel, estradiol benzoate, estradiol hemihydrate, and estradiol valerate) or synthetic estradiol (typically not detectable by assay: ethinyl estradiol and mestranol)^6,21–23^.

### Longitudinal study

Conducted in BKK, this cohort enrolled 25 TGW planning FHT initiation (FHT-naïve or off FHT at least six months). After loss to follow-up (*n* = 3) and withdrawal (*n* = 1), 21 participants were included.

### Study Procedures

#### Cross-sectional

Participants completed two visits: screening (informed consent, sexual and medical history, medication review, physical examination, rapid HIV testing, safety labs) and sampling three days to 12 weeks later. Rectal mucosal (RM) secretions were collected with polyurethane swabs using sigmoidoscopy or anoscopy. Serum estradiol and total testosterone concentrations were measured. TGW with vaginoplasty could undergo optional neovaginal sampling using a speculum and polyurethane swab.

#### Longitudinal

Participants completed three visits: screening as above, followed by pre-FHT sampling one day to two weeks later, and post-FHT sampling three to 12 months after FHT initiation, with RM and serum hormone collection at each sampling visit.

### Serum hormone measurements

Serum estradiol and testosterone concentrations were measured at all sampling visits. For the Atlanta cohort, serum was collected via SST tubes and cryopreserved for batch analyses. Serum hormone concentrations were quantified via Estradiol (DRG, #75871-318) and Testosterone (DRG, #75871-120) ELISA kits, according to manufacturer instructions. For the BKK cohorts, sex hormones were quantified using a standard clinical immunoassay. A subset of BKK serum samples (*n* = 41) was also analyzed with the DRG ELISAs in Atlanta; intraclass correlation coefficients (ICC) for estradiol (ICC = 0.86) and testosterone (ICC = 0.92) indicated intertest (lab-based ELISA vs clinical immunoassay) reliability.

### Microbiota sequencing

RM swabs were collected once from 205 participants in the cross-sectional study and twice in 21 longitudinal participants before and after FHT initiation. Neovaginal swabs were collected in a subset of 13 BKK TGW. To minimize the potential for batch effect, all specimens were extracted at Emory University, and sequencing was conducted in two batches that contained roughly equal distributions of MSM, TGW, ATL, and BKK participants. DNA was extracted using the Qiagen DNeasy PowerSoil Pro Kit. Libraries were made using a modification of the Illumina 16S Metagenomic Sequencing Library Preparation workflow. 12.5 ng of DNA was amplified using 16S Amplicon PCR Forward and Reverse Primers. Libraries were purified with Ampure XP beads. Purified amplicons were indexed with Nextera XT Index primers, and indexed amplicons were purified with Ampure XP beads. Final 16S libraries were approximately 630 bp and were pooled in equal amounts based on fluorescence quantification. Final library pools were quantitated via qPCR. The pooled library was sequenced on an Illumina MiSeq using MiSeq v3 600 cycle chemistry at a loading density of 6–8 pM with 20% PhiX, generating roughly 20 million, 300 bp paired-end reads. Recommended sequencing depth was >100,000 reads per sample. Raw amplicon sequence reads were evaluated for quality control (QC) using FastQC v.0.12.0 and Fastq_Screen v.0.15.3 with MultiQC v1.22.3^25,26^ and were then processed using Quantitative Insights into Microbial Ecology (QIIME2 v2023.7)^27^. The Divisive Amplicon Denoising Algorithm 2 (DADA2) package^28^ was used within QIIME2 to denoise and dereplicate all paired-end sequences and to create the feature table of amplicon sequence variants used within QIIME2. DADA2 parameters were chosen to trim the first 30bp, to truncate both paired-end reads at position 240, and to remove chimeric reads. Taxonomic assignment was performed via QIIME2, and the data were aligned to Silva (v132.1)^29^ using the QIIME taxonomy modules. To monitor potential contamination during laboratory processing, negative controls were included throughout the workflow. Extraction blank controls containing all reagents but no biological material were processed alongside the study samples during DNA extraction using the Qiagen DNeasy PowerSoil Pro Kit. PCR negative controls containing nuclease-free water in place of template DNA were included during amplification. These controls were carried through library preparation and sequenced together with the study samples. Sequencing reads from negative controls were evaluated during quality control and downstream bioinformatic processing to assess potential reagent or laboratory contamination. Standard contamination mitigation practices were followed throughout laboratory processing, including the use of sterile consumables, separation of pre- and post-PCR work areas, and routine decontamination of surfaces and equipment.

### Statistics and reproducibility

#### Demographic and hormone analyses

Group differences were assessed using Kruskal-Wallis tests for continuous variables and Fisher’s exact tests for categorical variables. Comparisons of FHT regimens and duration categories in Table 2 used similar nonparametric approaches (Kruskal-Wallis or Wilcoxon where appropriate) for continuous measures and Fisher’s exact tests for categorical variables, including regimen classifications and treatment duration groupings.

For cross-sectional serum hormone analyses, differences in the proportions of ATL TGW and BKK TGW demonstrating therapeutic estradiol and testosterone concentrations (as defined by Endocrine Society Guidelines) were assessed using Fisher’s exact tests. Individual cross-sectional hormone concentrations were also examined, given the use of different immunoassays at the two sites and the potential for reduced agreement at extreme values. Despite high intraclass correlation coefficients, these values were not considered directly comparable between ATL and BKK for primary analyses. For longitudinal analyses of serum hormone concentrations in BKK TGW, within-participant changes over time were assessed using Wilcoxon tests. All statistical tests were two-tailed, with statistical significance defined as *p* < 0.05. Analyses were conducted using GraphPad Prism. The lowest limit of *p*-value calculation in GraphPad Prism is <0.0001.

#### Microbiota analyses

Using linear decomposition modeling (LDM)^30^ and BOUTH analyses^31^ described further below, RM microbiota were compared by gender category (ATL TGW vs MSM; BKK TGW vs MSM) and geography (TGW ATL vs BKK; MSM ATL vs BKK) for the cross-sectional study. Rectal and neovaginal microbiota were also compared among a subset of BKK TGW. For the longitudinal study, RM microbiota before and after FHT initiation were compared. We employed complementary LDM and BOUTH approaches in this study to both identify highly significant correlations with individual taxa and variables of interest using a statistically rigorous method (LDM) and to identify which of these individual taxa were the most significant contributors to the observed differences using BOUTH.

Microbiota composition was assessed globally and at the level of individual taxa. Alpha diversity (Shannon index) was compared using the Wilcoxon test. Relative abundance and presence-absence data were analyzed using LDM, which is a robust statistical approach that allows the inclusion of multiple study groups and visits into a single model, controls for multiple comparisons, and is a single analysis pathway that combines both a global test of effect as well as tests of individual outcomes^30,32^. The LDM essentially fits a linear model for taxon relative abundance or presence-absence data as the outcome and regresses it on continuous traits or categorical group variables, while adjusting for potential confounders; however, it differs from standard linear regression in that it uses permutation-based p-values to account for non-normally distributed data. For these analyses, data were normalized to have a mean of zero and a standard deviation of 1; missing data were imputed by the mean of the observed data.

Presence-absence analyses were included to ensure the detection of rare differences in the rectal microbiome, as they are the preferred method of rare taxa detection^32^. Many associations are driven by changes in which taxa are present and which are absent, however confounding by read depth is a limitation to standard relative abundance analysis. Our method of evaluating presence-absence involves first rarifying the ASV table such that all samples have the same library size, which eliminates confounding by read depth, and then repeatedly applying the LDM to all (i.e., infinitely) rarified taxa count tables^33^. Analyses were conducted at the genus level.

We next applied Bottom-Up Approach to Testing Hypotheses (BOUTH analysis) to the cross-sectional relative abundance data to identify the “driver taxa” contributing most strongly to group differences. Bottom-up approaches are better suited to detecting signals within the phylogenetic tree, as microbiome data tend to fall at varying levels of taxonomic depth. BOUTH evaluates associations starting at the lowest phylogenetic level and aggregates evidence upward to identify the taxonomic groups that most influence community structure while controlling a novel error rate called the false selection rate. The direction of effect may be different for each of the lower-level taxa that together contribute to the enriched signal, but we were able to estimate the overall direction of effect using relative abundance LDM at the enriched phylogenetic level. This framework improves interpretability by highlighting biologically meaningful driver taxa rather than large sets of individually significant but difficult-to-contextualize associations. Simulation studies and empirical applications consistently show that BOUTH more effectively isolates true “driver taxa” than conventional methods, which often overlook tree-structured dependencies or fail to aggregate related signals^31^.

For both the LDM and BOUTH analyses, we included batch and, where appropriate, location as covariates. We did not include behavioral covariates in the main analyses because many (PrEP use, sexual practices, douching, etc.) are correlated with one another and closely associated with the primary grouping variables (gender identity and study site). Including them could result in overadjustment, as these variables may be conceptually intertwined with, or potentially on the causal pathway of, the exposures of interest. We conducted additional sensitivity analyses where we repeated all cross-sectional LDM and BOUTH analyses, adjusting for RAI frequency, PrEP use, prior STI, douching, tobacco use, alcohol use, and drug use covariates to assess whether the directions of effect remained consistent with the initial analyses, which would indicate that these variables were unlikely to be responsible for significant differences between the groups.

Finally, LDM was conducted at the family level, which had fewer unidentified taxa than the genus level, to explore associations between serum estradiol and testosterone concentrations and microbiome composition. Because no significant associations were identified by LDM, we next used Spearman correlations at the family level that were not corrected for multiple comparisons to assess associations for exploratory/hypothesis-generating purposes.

Global significance was defined at *p* < 0.05, with FDR of 5% for individual taxa, in LDM analyses.

### Reporting summary

Further information on research design is available in the Nature Portfolio Reporting Summary linked to this article.

## Results

### The clinical cohort

Demographic and clinical characteristics are summarized in Table 1. The median age was higher in ATL MSM (34 years) than among BKK MSM and TGW in both cities (26 years). The ATL population was racially diverse and representative of the local community. Pre-exposure prophylaxis (PrEP) use was highest in ATL MSM (60.6%) compared with ATL TGW (24%), BKK MSM (32%), and BKK TGW (10.3%). Recent RAI was most common in BKK TGW, followed by BKK MSM, ATL MSM, and ATL TGW. Douching before sex was reported more commonly in BKK participants (TGW 86.6%, MSM 92%) than in ATL participants (TGW 56%, MSM 60.6%). Tobacco, alcohol, and drug use were also more frequently reported in ATL than in BKK participants.

**Table 1:** Demographic data

	Cross-sectional cohort					Longitudinal cohort
	Atlanta		Bangkok			Bangkok
Characteristic	TGW (*n* = 25)	MSM (*n* = 33)	TGW (*n* = 97)	MSM (*n* = 50)	*P* value	TGW (*n* = 21)
Age in years; median (IQR)	26 (24–33)	34 (28–38)	26 (23–32)	26 (24–28)	0.0002	28 (24–34)
Race; n (%)						
Black	8 (32)	15 (45.5)	0 (0)	0 (0)	N/A	0 (0)
White	14 (56)	13 (39.4)	0 (0)	0 (0)		0 (0)
Asian	2 (8)	5 (15.2)	97 (100)	50 (100)		21 (100)
Other	1 (4)	0 (0)	0 (0)	0 (0)		0 (0)
# of RAI events in prior 3 months; median (IQR)	1 (0–2)	2 (0.5–4.5)	5 (2–10)	3 (2–8.5)	0.0001	5 (2.25–8.25)
PrEP use; n (%)	6 (24)	20 (60.6)	10 (10.3)	16 (32)	<0.0001	5 (23.8)
Lubricant use; n (%)	22 (88)	30 (90.9)	88 (90.7)	49 (98)	0.27	20 (95.2)
Douche before sex; n (%)	14 (56)	20 (60.6)	84 (86.6)	46 (92)	<0.0001	17 (81.0)
Self-reported prior STI; n (%)	6 (24)	18 (54.6)	9 (9.3)	16 (32)	<0.0001	2 (9.5)
Tobacco in prior 6 months; n (%)	9 (36)	9 (27.3)	12 (12.4)	6 (12)	0.02	2 (9.5)
Current alcohol use; n (%)	20 (80)	25 (75.8)	31 (32)	9 (18)	<0.0001	6 (28.6)
Drug use in prior 6 months; n (%)	12 (48)	12 (36.4)	6 (6.2)	0 (0)	<0.0001	2 (9.5)

*TGW* transgender women who have sex with men, *MSM* cisgender men who have sex with men, *RAI* receptive anal intercourse, *PrEP* pre-exposure prophylaxis, *STI* sexually transmitted infection, *IQR* interquartile range. Statistical comparisons were made with a two-sided Kruskal-Wallis test for continuous variables (Age, # of RAI events in prior 3 months) and a two-sided Fisher’s exact test for discrete variables (Race, PrEP use, Lubricant use, Douche use, Self-reported prior STI, Tobacco use, Alcohol use, Drug use).

### Feminizing hormone therapy regimens

#### Cross-sectional cohort

FHT regimens and durations are presented in Table 2. Compared with ATL TGW, BKK TGW more frequently used synthetic estradiol (ethinyl estradiol or mestranol). Although the use of anti-androgens and progestins did not differ significantly between groups, duration of therapy was significantly longer among BKK participants in both the anti-androgen- and progestin-treated subgroups. Among ATL TGW, spironolactone was the predominant anti-androgen, whereas all BKK TGW used cyproterone acetate. Progestin formulations included hydroxyprogesterone caproate, progesterone, levonorgestrel, and norethisterone. Cyproterone acetate has some progestin activity; however, given that its primary clinical use is as an anti-androgen, we categorized it as anti-androgen only.

**Table 2:** Feminizing hormone therapy (FHT) regimens and durations for the cross-sectional cohort

	Cross-Sectional Cohort		
FHT Regimen	Atlanta TGW (n = 25)	Bangkok TGW (n = 97)	P value
Synthetic estradiol; n (%)	0 (0)	22 (22.7)	0.007
Months on estradiol; median (IQR)	20.5 (12–37)	85 (41–120)	0.0002
Anti-androgen; n (%)	18 (72)	59 (60.8)	0.36
Months on anti-androgen; median (IQR)*	19 (12–27)	79 (42–109)	<0.0001
Progestin; n (%)	11 (44)	42 (43.3)	1.0
Months on progestin; median (IQR)*	12 (5.75–24)	97 (48–143)	<0.0001

*TGW* transgender women who have sex with men, *MSM* cisgender men who have sex with men. Statistical comparisons were made with a two-sided Wilcoxon test for continuous variables and a two-sided Fisher’s exact test for discrete variables. Synthetic estradiol formulations included ethinyl estradiol and mestranol. For ATL TGW, spironolactone comprised most anti-androgen formulations, with one participant taking cyproterone acetate. For BKK TGW, 100% of anti-androgen formulations were cyproterone acetate. Progestin formulations included hydroxyprogesterone caproate, progesterone, levonorgestrel, and norethisterone. *For the months on anti-androgen and progestin, we limited analysis to those who reported use of these medications.

#### Longitudinal cohort

FHT regimens and duration are summarized in Table 3. Patterns of synthetic estradiol and anti-androgen use were similar to BKK TGW in the cross-sectional cohort, though only ethinyl estradiol was used in the longitudinal cohort. All anti-androgen formulations were cyproterone acetate, and no participants were taking progestins. Participants completed post-FHT sampling a median of 120 days (IQR 96–182) after estradiol initiation.

**Table 3:** Feminizing hormone therapy (FHT) regimens and durations for the Longitudinal Cohort

Longitudinal Cohort - Bangkok TGW (n = 21)	
FHT Regimen	
Synthetic estradiol; n (%)	4 (19.1)
17β estradiol; n (%)	18 (85.7)
Days between estradiol initiation and post-FHT sampling; median (IQR)	120 (96–182)
Anti-androgen; n (%)	15 (61.9)
Days between anti-androgen initiation and post-FHT sampling; median (IQR)	99 (0–158)
Progestin; n (%)	0 (0)

*TGW* transgender women who have sex with men, *MSM* cisgender men who have sex with men. 100% of synthetic estradiol formulations were ethinyl estradiol. 17β estradiol formulations include 17β-estradiol gel, estradiol benzoate, estradiol hemihydrate, and estradiol valerate. 13/15 participants with anti-androgen activity were taking anti-androgen medication, and an additional 2/15 had orchiectomy prior to study initiation. 100% of anti-androgen formulations were cyproterone acetate.

### Serum hormone concentrations

**Fig. 2:**
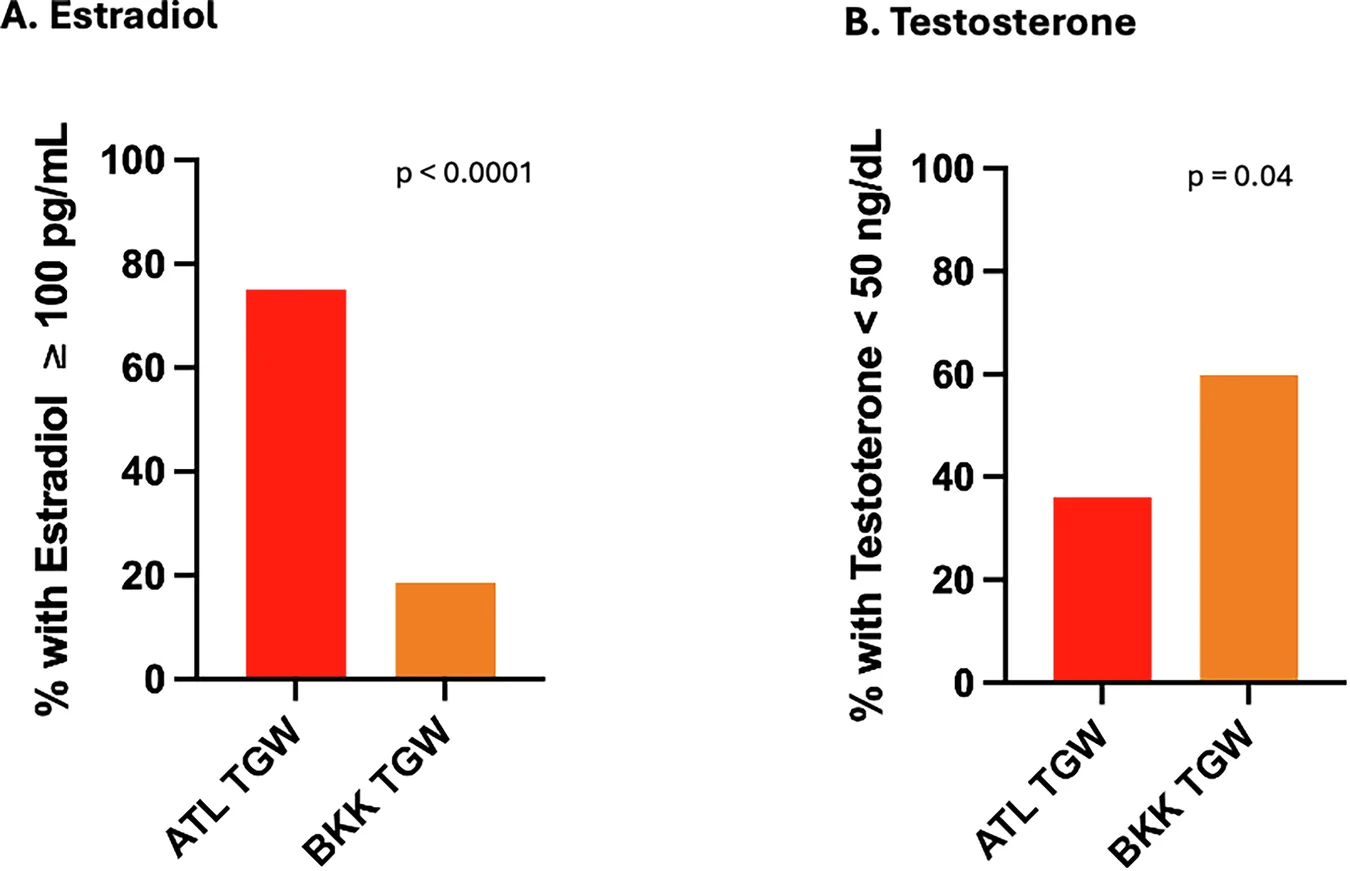
Proportions of ATL TGW versus BKK TGW participants who demonstrated therapeutic serum hormone concentrations. These plots display the percentage of ATL TGW (*n* = 25) and BKK TGW (*n* = 97) who demonstrated therapeutic serum estradiol (**A**) and testosterone (**B**) concentrations. Therapeutic concentrations are defined as ≥ 100 pg/mL for serum estradiol and <50 ng/dL for serum testosterone per Endocrine Society Guidelines. For ATL TGW, 19/25 (76%) demonstrated therapeutic serum estradiol concentrations, and 9/25 (36%) demonstrated therapeutic serum testosterone concentrations. For BKK TGW, 18/97 (18.6%) demonstrated therapeutic serum estradiol concentrations, and 58/97 (59.8%) demonstrated therapeutic serum testosterone concentrations. Between-group comparisons were conducted with two-sided Fisher’s exact tests.

The proportions of TGW demonstrating therapeutic serum hormone concentrations differed between sites (Fig. 2). In ATL, 76% of TGW demonstrated therapeutic estradiol concentrations and 36% demonstrated therapeutic testosterone suppression, compared with 18.6% and 59.8%, respectively, in BKK TGW. Therapeutic estradiol was defined as ≥ 100 pg/mL and therapeutic testosterone as <50 ng/dL, in accordance with Endocrine Society Guidelines^34^.

Descriptive comparisons of cross-sectional hormone distributions showed higher median estradiol concentrations in ATL TGW (189 pg/mL) than in BKK TGW (24 pg/mL), whereas median testosterone concentrations were lower in BKK TGW (24.5 ng/dL) than in ATL TGW (74 ng/dL). However, given the use of different immunoassays across sites and reduced reliability of agreement at extreme values, these cross-site comparisons should be interpreted with caution. Individual cross-sectional hormone concentrations are presented in Supplementary Fig. 1.

**Fig. 3:**
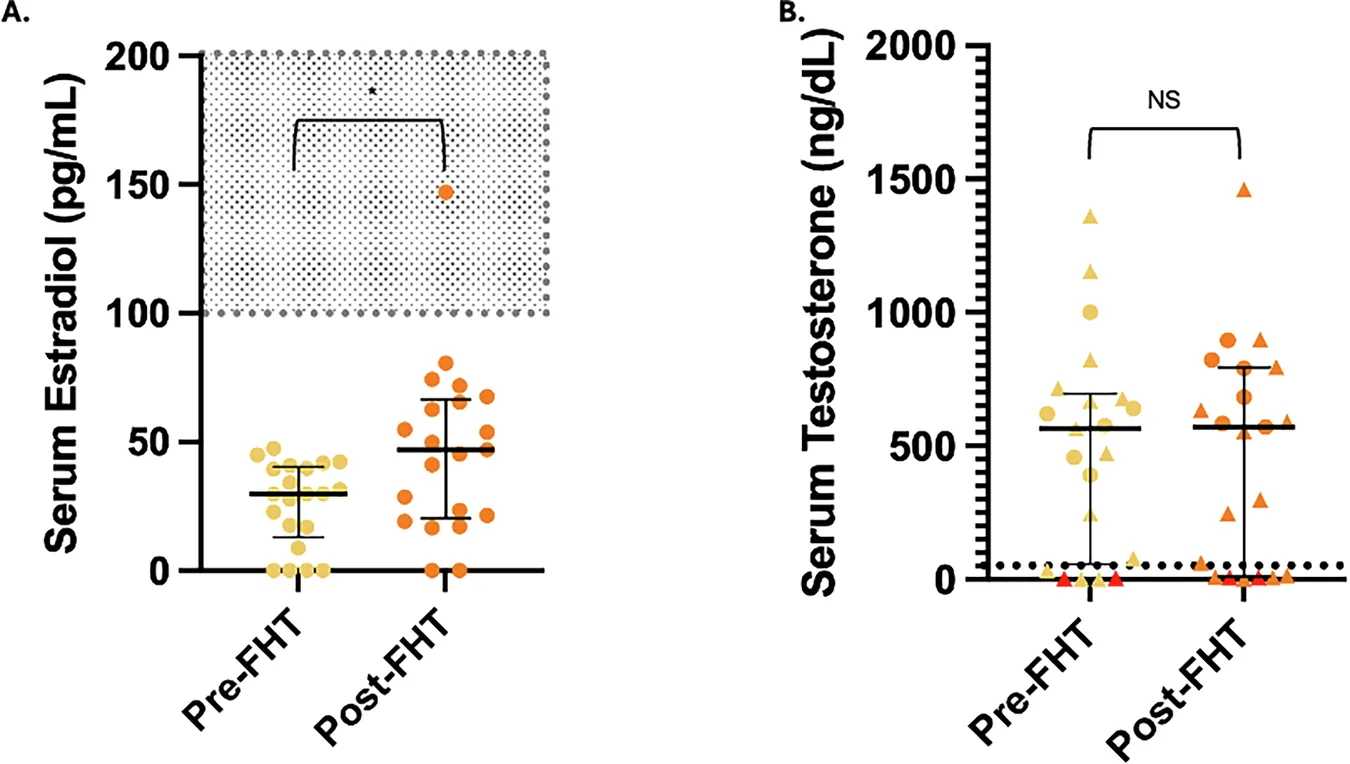
Longitudinal serum hormone concentrations. These plots display serum estradiol (**A**) and testosterone (**B**) concentration medians and interquartile ranges in the longitudinal study group before and 3–12 months after feminizing hormone therapy (FHT) initiation (*n* = 21). 13/21 participants initiated anti-androgen therapy as part of their FHT; these are indicated by orange triangles in 2B. 2/21 additional participants had undergone orchiectomy prior to study initiation; these are indicated by red triangles in 2B. The shaded area (estradiol) and dotted line (testosterone) indicate therapeutic concentrations for TGW per Endocrine Society Guidelines (100–200 pg/mL for serum estradiol and <50 ng/dL for serum testosterone). Comparisons were conducted with two-sided Wilcoxon tests (Estradiol Pre-FHT vs Post-FHT *p* = 0.02; Testosterone Pre-FHT vs Post-FHT *p* = 0.97). * = *p* < 0.05.

Longitudinal serum hormone concentrations are shown in Fig. 3. While median estradiol increased significantly (*p* = 0.02) from pre-FHT (29.8 pg/mL) to post-FHT (46.9 pg/mL), the effect size was small, and the post-FHT median estradiol remained well below the lower limit of the therapeutic window for this hormone. Median testosterone concentration did not change significantly after FHT initiation and remained persistently above the therapeutic window^34^.

### Rectal microbiota differences by gender category and geography

Table 4 summarizes mucosal microbiota alpha and beta diversity comparisons.

**Table 4:** Summary of mucosal microbiota comparisons

		α Diversity		β Diversity			
Type of Comparison	Group Comparison	Median Shannon Index	P value	Relative Abundance LDM Global p	# Differentially Abundant Taxa, FDR < 5%	Presence-Absence LDM Global p	# Taxa w/ Differential Probability of Presence, FDR < 5%
Gender Category	ATL: TGW vs MSM	2.82 vs 3.13	0.12	0.004	21 (13 ↑ in MSM; 8 ↑ in TGW)	0.0002	19 (12 ↑ in MSM; 7 ↑ in TGW)
	BKK: TGW vs MSM	2.67 vs 2.61	0.96	0.005	0 (3 w/ FDR < 20%; 2 ↑ in MSM; 1 ↑ in TGW)	0.03	0 (4 < 20%; 3 ↑ in MSM; 1 ↑ in TGW)
Geographic Location	TGW: ATL vs BKK	2.82 vs 2.67	0.052	0.002	20 (16 ↑ in ATL; 4 ↑ in BKK)	0.0006	8 (3 ↑ in ATL; 5 ↑ in BKK)
	MSM: ATL vs BKK	3.13 vs 2.61	0.002	0.0002	66 (55 ↑ in ATL; 11 ↑ in BKK)	0.0002	64 (50 ↑ in ATL; 14 ↑ in BKK)
Mucosal Site	BKK TGW: Rectal vs Neovaginal	2.65 vs 2.18	0.0004	0.0002	88 (60 ↑ in rectum; 28 ↑ in neovagina)	0.0002	86 (70 ↑ in rectum; 16 ↑ in neovagina)
Pre/Post FHT	BKK TGW: Pre- vs Post-FHT	2.82 vs 2.61	1	0.8	0	0.8	0

*RA* relative abundance analysis, *LDM* linear decomposition modeling, *FDR* false discovery rate, *PA* presence-absence analysis, *FHT* feminizing hormone therapy, *ATL* Atlanta, Georgia, USA, *BKK* Bangkok, Thailand, *TGW* transgender women who have sex with men, *MSM* cisgender men who have sex with men. Median Shannon Indices were compared with a two-sided Wilcoxon test.

#### Atlanta

**Fig. 4:**
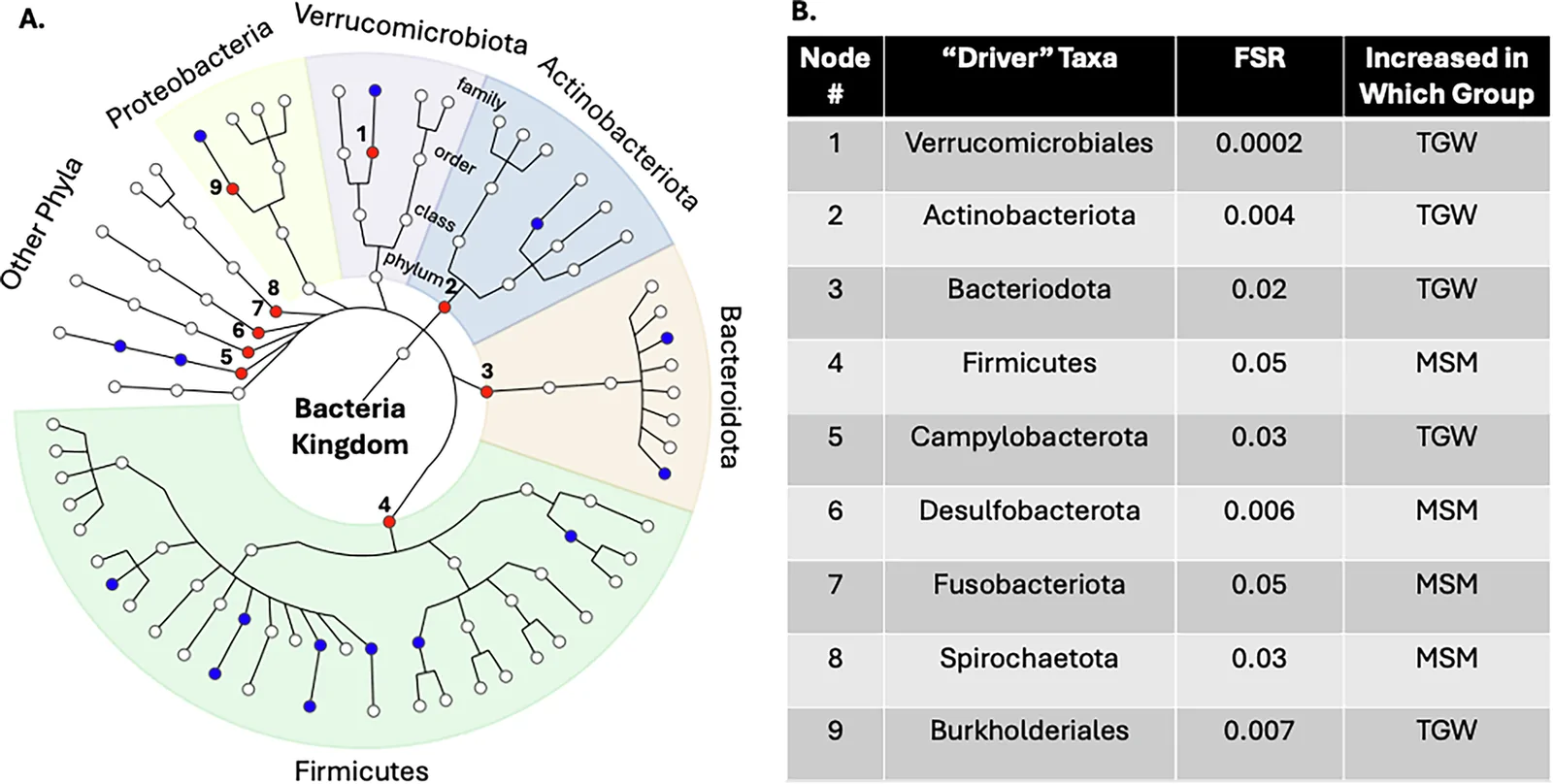
“Drivers” of gender category differences in Atlanta. We first conducted linear decomposition modeling (LDM) of the rectal microbiota relative abundance data comparing ATL TGW (transgender women in Atlanta; *n* = 25) and ATL MSM (cisgender men who have sex with men in Atlanta; *n* = 33) at the genus level. Statistical significance for the global test was assessed using a permutation-based LDM test (two-sided), yielding a significant difference (*p* = 0.004). We then used the Bottom-Up Approach to Testing Hypotheses (BOUTH) analysis of this LDM data to identify the “driver taxa,” with control for multiple comparisons using the false selection rate (FSR) < 0.05, which represent the highest-level taxa below which there are dense association signals. **A** Demonstrates the BOUTH plot with the nine driver taxa numbered and represented with red circles and significantly different non-driver taxa represented with blue circles. The circle in the middle of the plot represents the *Bacteria* kingdom, and each concentric circle of taxa represents the subsequent phylogenetic level (phylum, class, order, family from center to periphery). The most abundant five phyla are identified with different colors and labeled around the circumference of the circle. **B** Lists the nine “driver taxa” for the significant difference between ATL TGW and ATL MSM, the FSR, and the direction of effect.

TGW and MSM had significantly different rectal microbiota compositions (LDM global *p* = 0.004 for relative abundance; LDM global *p* = 0.0002 for presence-absence). Twenty-one taxa differed by relative abundance and 19 by presence-absence (FDR < 5%; Supplementary Fig. 2). BOUTH analysis (Fig. 4) identified “driver taxa” including seven phyla: *Actinobacteriota, Bacteroidota*, and *Campylobacterota* (enriched in TGW) and *Firmicutes, Desulfobacterota, Fusobacteriota*, and *Spirochaetota* (enriched in MSM); and two orders: *Verrucomicrobiales* and *Burkholderiales* (both enriched in TGW). Alpha diversity did not differ by gender category in ATL (*p* = 0.12).

#### Bangkok

**Fig. 5:**
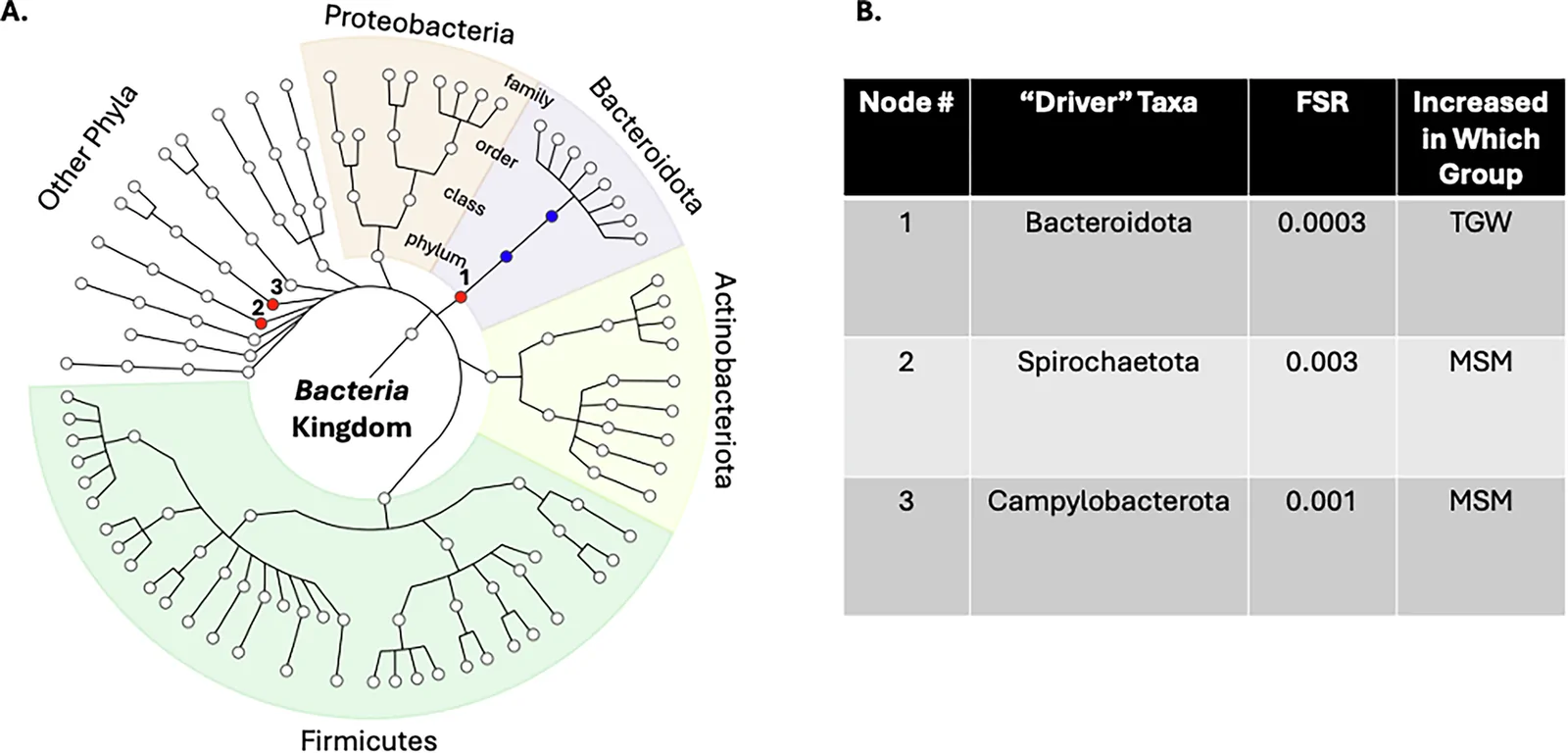
“Drivers” of gender category differences in Bangkok. We first conducted linear decomposition modeling (LDM) of the rectal microbiota relative abundance data comparing BKK TGW (transgender women in Bangkok; *n* = 97) and BKK MSM (cisgender men who have sex with men in Bangkok; *n* = 50) to the genus level. Statistical significance for the global test was assessed using a permutation-based LDM test (two-sided), yielding a significant difference (*p* = 0.005). We then used the Bottom-Up Approach to Testing Hypotheses (BOUTH) analysis of this LDM data to identify the “driver taxa,” with control for multiple comparisons using the false selection rate (FSR) < 0.05, which represent the highest-level taxa below which there are dense association signals. **A** Demonstrates the BOUTH plot with the three driver taxa numbered and represented with red circles and significantly different non-driver taxa represented with blue circles. The circle in the middle of the plot represents the *kingdom Bacteria*, and each concentric circle of taxa represents the subsequent phylogenetic level (phylum, class, order, family, from the center to the periphery). The most abundant five phyla are identified with different colors and labeled around the circumference of the circle. **B** Lists the three “driver taxa” for the significant difference between BKK TGW and BKK MSM, the FSR, and the direction of effect.

TGW and MSM also showed significant global differences (LDM global *p* = 0.005 for relative abundance, LDM global *p* = 0.03 for presence-absence). No individual taxa reached FDR < 5%. In exploratory analyses utilizing a more liberal FDR of <20%, three taxa differed in relative abundance and four in presence-absence (Supplementary Fig. 3). BOUTH identified three driver phyla: *Bacteroidota* (again enriched in TGW) and *Spirochaetota* and *Campylobacterota* (enriched in MSM; Fig. 5). Alpha diversity did not differ by gender category in BKK (*p* = 0.96).

#### Transgender women

**Fig. 6:**
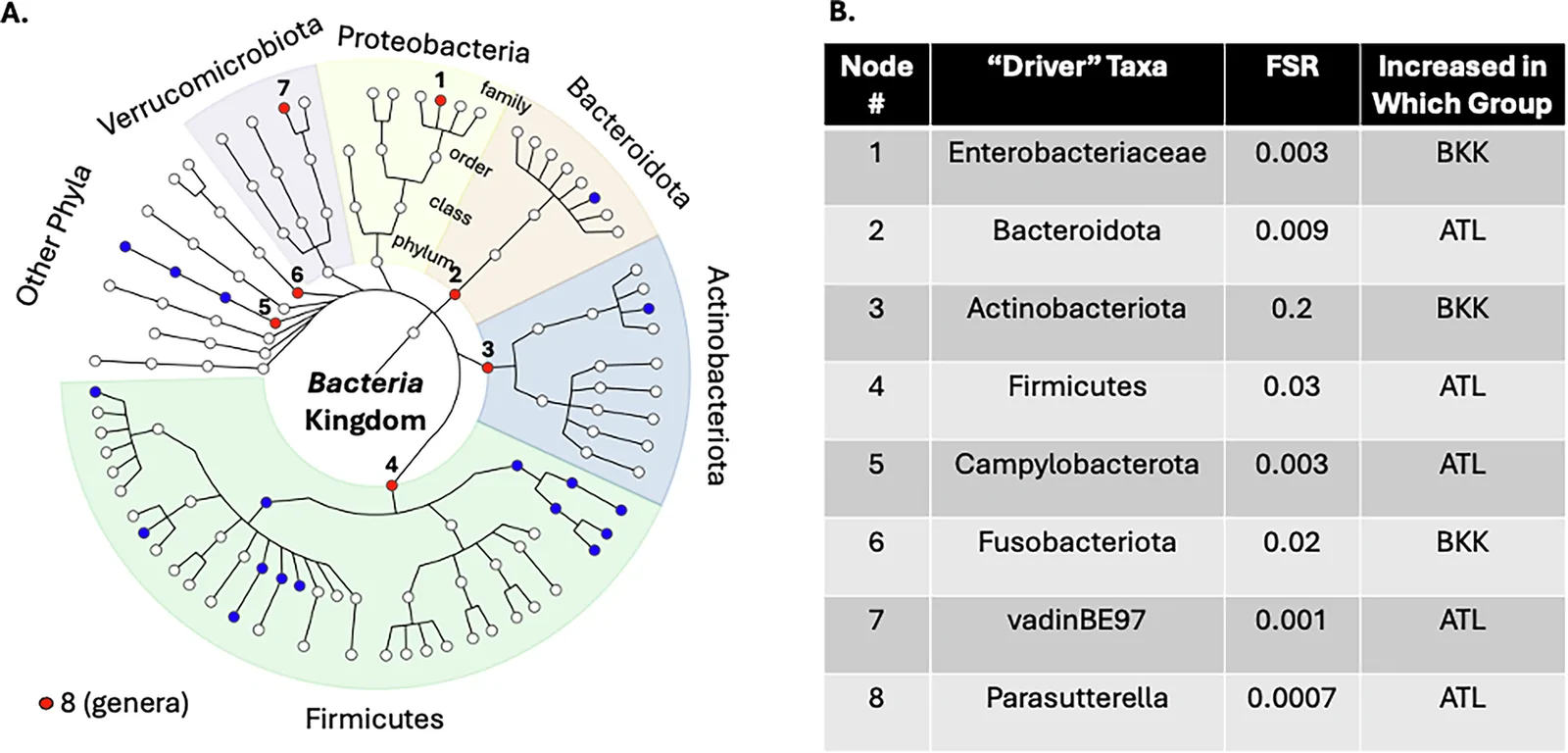
“Drivers” of location differences within transgender women. We first conducted linear decomposition modeling (LDM) of the rectal microbiota relative abundance data comparing BKK TGW (transgender women in Bangkok; *n* = 97) and ATL TGW (transgender women in Atlanta; *n* = 25) to the genus level. Statistical significance for the global test was assessed using a permutation-based LDM test (two-sided), yielding a significant difference (*p* = 0.002). We then used the Bottom-Up Approach to Testing Hypotheses (BOUTH) analysis of this LDM data to identify the “driver taxa,” with control for multiple comparisons using the false selection rate (FSR) < 0.05, which represent the highest-level taxa below which there are dense association signals. **A** Demonstrates the BOUTH plot with the eight driver taxa numbered and represented with red circles and significantly different non-driver taxa represented with blue circles. The circle in the middle of the plot represents the *Bacteria* kingdom, and each concentric circle of taxa represents the subsequent phylogenetic level (phylum, class, order, family from center to periphery). The most abundant five phyla are identified with different colors and labeled around the circumference of the circle. **B** Lists the eight “driver taxa” for the significant difference between BKK TGW and ATL TGW, the FSR, and the direction of effect.

Rectal microbiota differed significantly between TGW in ATL and BKK (LDM global *p* = 0.002 for relative abundance, LDM global *p* = 0.0006 for presence-absence). Twenty taxa differed by relative abundance and eight by presence-absence (FDR < 5%; Supplementary Fig. 4). BOUTH identified eight driver taxa: five phyla (*Bacteroidota, Firmicutes, Campylobacterota* enriched in ATL TGW (which were also enriched in ATL MSM, discussed below); *Actinobacteriota, Fusobacteriota* enriched in BKK TGW), two families (*vadinBE97* enriched in ATL TGW; *Enterobacteriaceae* enriched in BKK TGW (which was also enriched in BKK MSM, discussed below)), and one genus (*Parasutterella*, enriched in ATL TGW; Fig. 6). Alpha diversity did not significantly differ by study site among TGW (*p* = 0.052).

#### Cisgender MSM

**Fig. 7:**
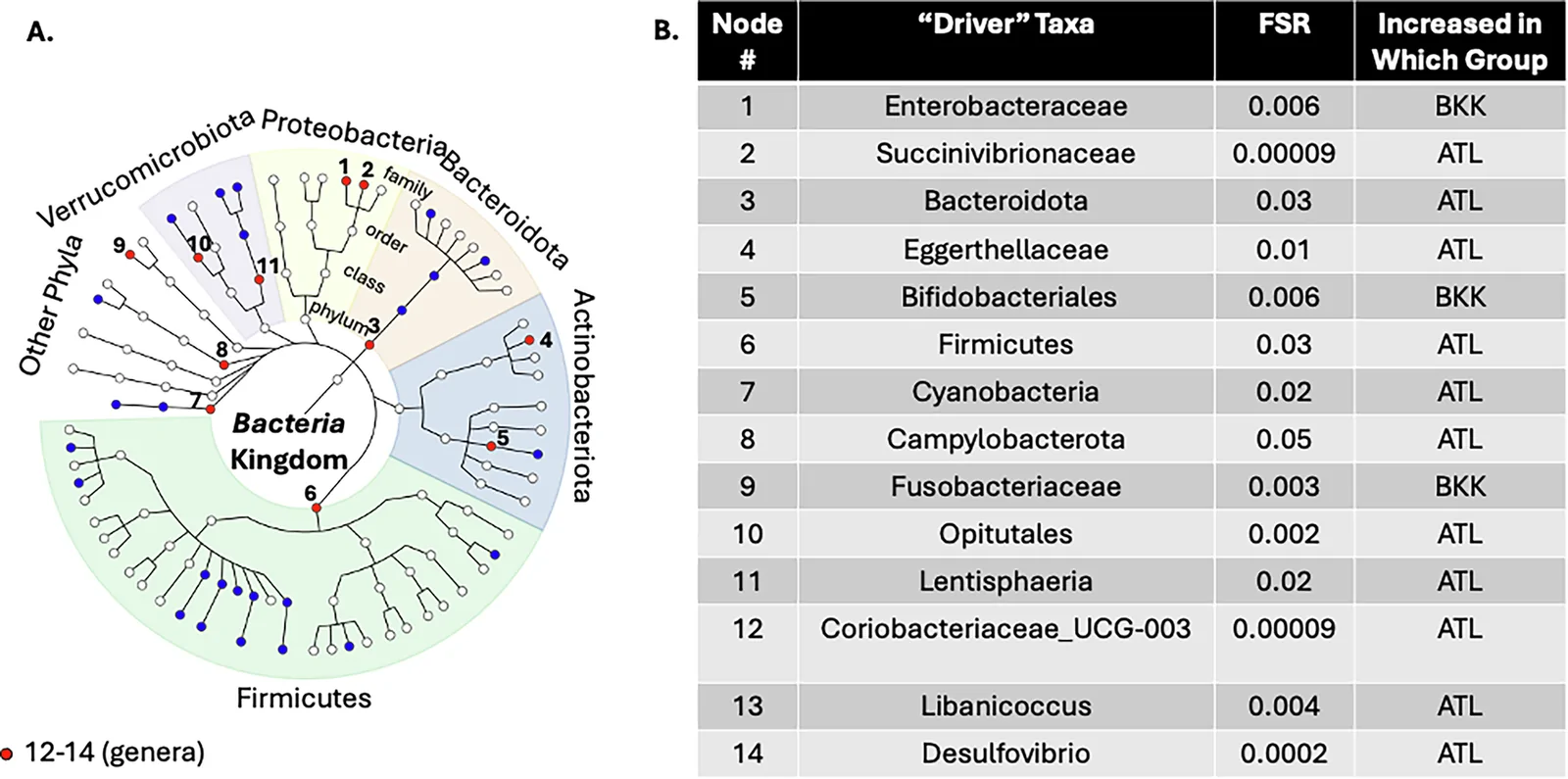
“Drivers” of location differences within cisgender men who have sex with men. We first conducted linear decomposition modeling (LDM) of the rectal microbiota relative abundance data comparing BKK MSM (cisgender men who have sex with men in Bangkok; *n* = 50) and ATL MSM (cisgender men who have sex with men in Atlanta; *n* = 33) to the genus level. Statistical significance for the global test was assessed using a permutation-based LDM test (two-sided), yielding a significant difference (*p* = 0.0002). We then used the Bottom-Up Approach to Testing Hypotheses (BOUTH) analysis of this LDM data to identify the “driver taxa,” with control for multiple comparisons using the false selection rate (FSR) < 0.05, which represent the highest-level taxa below which there are dense association signals. **A** Demonstrates the BOUTH plot with the 14 driver taxa numbered and represented with red circles and significantly different non-driver taxa represented with blue circles. The circle in the middle of the plot represents the *Bacteria* kingdom, and each concentric circle of taxa represents the subsequent phylogenetic level (phylum, class, order, family from center to periphery). The most abundant five phyla are identified with different colors and labeled around the circumference of the circle. **B** Lists the 14 “driver taxa” for the significant difference between BKK MSM and ATL MSM, the FSR, and the direction of effect.

Rectal microbiota also differed significantly between MSM in ATL and BKK (LDM global *p* = 0.0002 for both relative abundance and presence-absence). Sixty-six taxa differed in relative abundance and 64 in presence-absence between MSM in ATL and BKK (FDR < 5%; Supplementary Fig. 5). BOUTH identified 14 driver taxa: four phyla (*Bacteroidota, Firmicutes, Campylobacterota, Cyanobacteria*—all enriched in ATL MSM); one class (*Lentisphaeria*, enriched in ATL MSM); two orders (*Opitutales*, enriched in ATL MSM; *Bifidobacteriales*, enriched in BKK MSM); four families (*Eggerthellaceae*—enriched in ATL MSM; *Succinivibrionaceae, Fusobacteriaceae, Enterobacteraceae*—enriched in BKK MSM (*Enterobacteraceae* also enriched in BKK TGW as above)); and three genera (*Coriobacteriaceae_UCG-003, Libanicoccus, Desulfovibrio*—all enriched in ATL MSM; Fig. 7). Alpha diversity was higher in ATL MSM compared to BKK MSM (*p* = 0.002).

### *Prevotellaceae* and *Bacteroidaceae* enrichment patterns

**Fig. 8:**
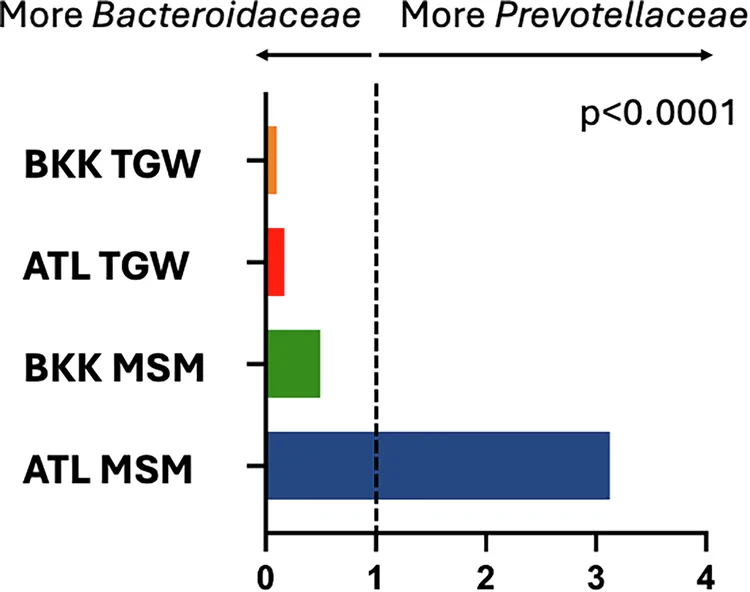
Rectal *Prevotellaceae* to *Bacteroidaceae* abundance ratios in key populations. Differences in rectal *Prevotellaceae* to *Bacteroidaceae* ratios across the four groups were assessed using a two-sided Kruskal-Wallis test, showing a significant overall difference (*p* < 0.0001). Post hoc pairwise comparisons were performed using Dunn’s test with Benjamini–Hochberg false discovery rate correction. BKK TGW = transgender women in Bangkok; ATL TGW transgender women in Atlanta, BKK MSM cisgender men who have sex with men in Bangkok, ATL MSM = cisgender men who have sex with men in Atlanta. The *Prevotellaceae:Bacteroidaceae* ratio was >1 for MSM in ATL but were <1 for MSM in BKK as well as TGW in both ATL and BKK.

To assess whether the pattern of *Prevotellaceae: Bacteroidaceae* enrichment seen in American and European MSM is generalizable to TGW and Asian cohorts, we compared the median *Prevotellaceae: Bacteroidaceae* abundance ratio across the four cross-sectional groups (BKK TGW, ATL TGW, BKK MSM, ATL MSM). Ratios differed significantly across the four groups (Kruskal-Wallis *p* < 0.0001). The median ratio was >1 in ATL MSM but <1 in BKK MSM and in TGW from both sites (Fig. 8).

We conducted sensitivity analyses for all cross-sectional LDM and BOUTH analyses, adjusting for seven behavioral covariates that differed significantly among the four groups: RAI frequency, PrEP use, prior STI, douching, tobacco use, alcohol use, and drug use. While statistical significance was attenuated, likely reflecting reduced power due to additional model complexity, multiple testing burden, and loss of observations from incomplete covariate data, the direction of effect for 100% of the original significantly different taxa remained unchanged, suggesting that the overall structure and directionality of the observed microbiome differences are robust and not predominantly due to differences in these seven behavioral factors.

### Rectal and neovaginal microbiota composition

**Fig. 9:**
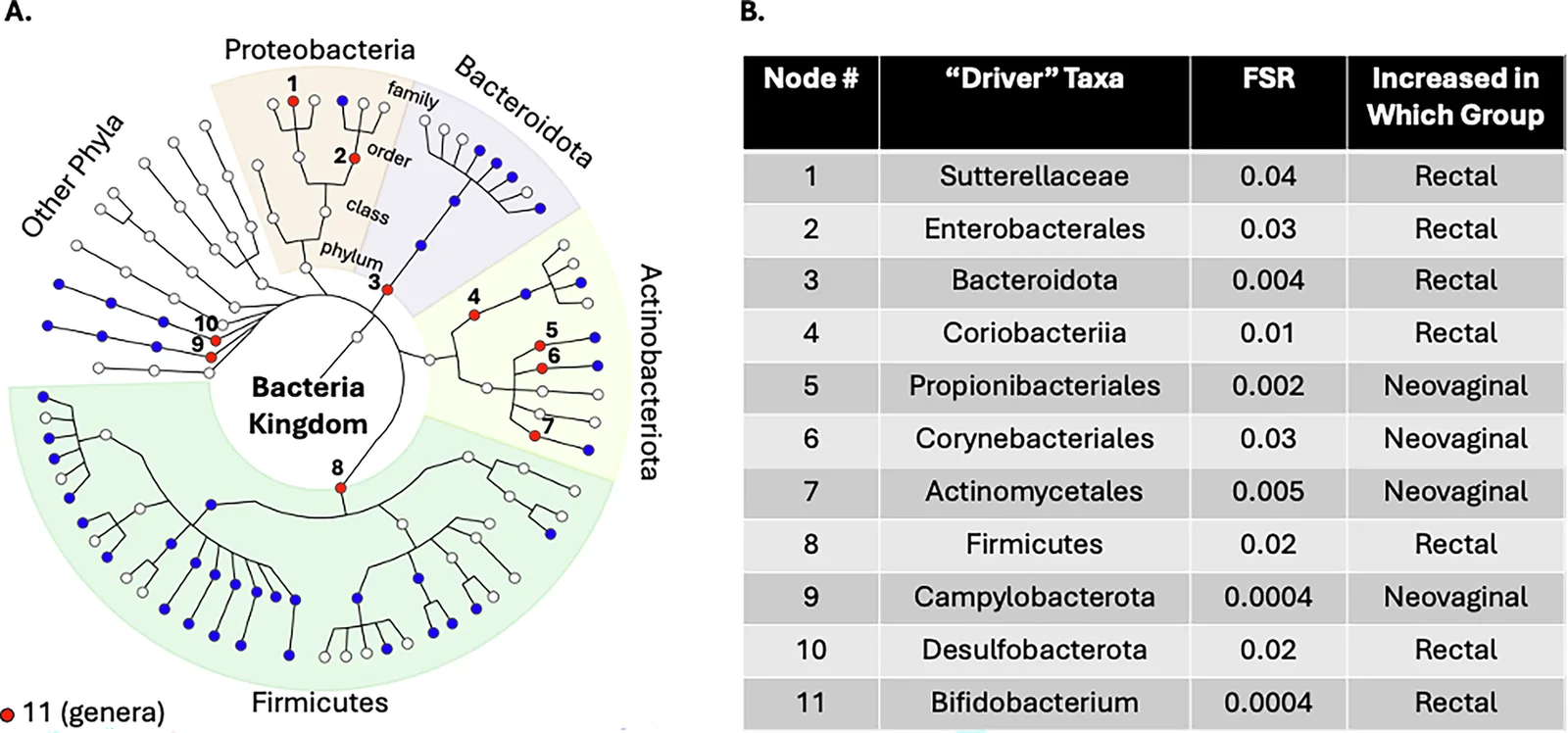
“Drivers” of rectal versus neovaginal mucosal site differences. We first conducted linear decomposition modeling (LDM) of the relative abundance data comparing the rectal and neovaginal microbiota in a subset of BKK TGW (transgender women in Bangkok; *n* = 97 with rectal sampling; *n* = 13 with neovaginal sampling) to the genus level. Statistical significance for the global test was assessed using a permutation-based LDM test (two-sided), yielding a significant difference (*p* = 0.0002). We then used the Bottom-Up Approach to Testing Hypotheses (BOUTH) analysis of this LDM data to identify the “driver taxa,” with control for multiple comparisons using the false selection rate (FSR) < 0.05, which represent the highest-level taxa below which there are dense association signals. **A** Demonstrates the BOUTH plot with the 11 driver taxa numbered and represented with red circles and significantly different non-driver taxa represented with blue circles. The circle in the middle of the plot represents the *Bacteria* kingdom, and each concentric circle of taxa represents the subsequent phylogenetic level (phylum, class, order, family from center to periphery). The most abundant five phyla are identified with different colors and labeled around the circumference of the circle. **B** Lists the 11 “driver taxa” for the significant difference between the rectal and neovaginal microbiota, the FSR, and the direction of effect.

We next compared the rectal (*n* = 97) and neovaginal (*n* = 13) microbiota in BKK TGW. Relative abundance and presence-absence both differed significantly (LDM global *p* = 0.0002 for each). Eighty-eight taxa differed by relative abundance and 86 by presence-absence (FDR < 5%; Supplementary Fig. 6 for taxa enriched in the neovagina and Supplementary Fig. 7 for taxa enriched in the rectum). BOUTH identified 11 driver taxa: four phyla (*Bacteroidota, Firmicutes, Desulfobacterota*—enriched in the rectum; *Campylobacterota*—enriched in the neovagina); one class (*Coriobacteriia*—enriched in the rectum); four orders (*Enterobacterales*—enriched in the rectum; *Propionibacteriales, Corynebacteriales, Actinomycetales*—enriched in the neovagina); one family (*Sutterellaceae*—enriched in the rectum); and one genus (*Bifidobacterium*—enriched in the rectum; Fig. 9). Alpha diversity was higher in the rectum compared to the neovagina (*p* = 0.0004).

Among participants with known neovaginal surgical technique (*n* = 12), nine had penile inversion and three had sigmoid colon pull-through. No significant differences in microbiota composition were observed between surgical types (LDM global *p* = 0.3), although the small number of sigmoid colon pull-through samples limited statistical power for this comparison.

### Associations between FHT and the rectal microbiota

We finally assessed associations with FHT and the rectal microbiota composition in two ways: first, by assessing for microbiota composition differences before and after FHT initiation in our longitudinal cohort, and second, by correlating serum hormone concentrations with microbiota relative abundance in our cross-sectional cohort.

No significant changes were observed before versus after FHT initiation for relative abundance, presence-absence, or alpha diversity (*p* = 0.8, 0.8, and 1, respectively).

Twenty-two of the 97 cross-sectional BKK TGW participants (22.7%) were taking synthetic estradiol, which is not detected by clinical serum estradiol assays. Consistent with this, participants on synthetic estradiol had lower measured estradiol concentrations than those on 17β estradiol formulations (Wilcoxon *p* = 0.001; Supplementary Fig. 8). Hence, we excluded these participants from the LDM assessing an association between serum estradiol and family-level microbiome relative abundance. This LDM, which included both the 75 remaining cross-sectional BKK TGW participants who were not taking synthetic estradiol and all 25 ATL TGW (*n* = 100), did not show a significant association (LDM global *p* = 0.6). The LDM assessing an association between serum testosterone and family-level microbiome relative abundance also showed no significant association (LDM global *p* = 0.6).

We subsequently conducted Spearman correlation analyses between serum hormone concentrations and family-level microbiome relative abundances in this limited subset to explore more subtle potential associations for hypothesis generation. These exploratory analyses were not adjusted for multiple comparisons. Using a nominal significance threshold of *p* < 0.05, we observed correlations between serum estradiol and six families and between serum testosterone and five families (Supplementary Tables 1 and 2).

## Discussion

We characterized rectal and neovaginal mucosal microbiota in TGW and MSM across Atlanta and Bangkok, identifying strong differences associated with both gender category and geography. Using the BOUTH method, we reduced large LDM-derived taxonomic signals to key “driver taxa” that best explained between-group variation, enabling the identification of microbial signatures specific to gender category and location. We also extended limited prior work on the neovaginal microbiota and identified enrichment of taxa overlapping with the BASIC cluster, previously linked to HIV seroconversion risk in mucosal sites^4,35^.

Interpretation of TGW-MSM differences must account for multiple overlapping biological and behavioral factors. In this study, TGW participants were receiving FHT and differed from MSM in several behaviors and clinical characteristics. Observed differences likely reflect combined biosocial influences rather than gender identity alone.

BOUTH analysis distilled numerous significant taxa into a focused set of drivers underlying group differences. We identified consistent enrichment of *Bacteroidota* in TGW across both sites. *Enterobacteriaceae* were enriched in BKK across both TGW and MSM, while ATL samples showed enrichment of *Bacteroidota, Firmicutes, and Campylobacterota*. These patterns suggest phylogenetically coherent microbial signatures influenced by both geography and gender identity.

Geographic effects were prominent and stable across gender identity. Higher *Enterobacteriaceae* in Bangkok is consistent with prior reports linking this family to dysbiosis, antibiotic exposure, and urbanization, including high regional prevalence of ESBL-producing strains^36^. In contrast, Atlanta-associated enrichment of *Bacteroidota, Firmicutes*, and *Campylobacterota* aligns with microbiota patterns previously linked to Western dietary exposures^37^. These findings reinforce the role of geography as a major determinant of rectal microbiota composition.

Within both locations, the gender category was strongly associated with rectal microbiota composition. BOUTH identified *Bacteroidota* as a key “driver” of differences between TGW and MSM in both cohorts, while *Firmicutes* was a driver in ATL MSM. These taxa align with prior literature linking microbial composition to sex hormone signaling. *Bacteroidota* includes taxa encoding enzymes involved in estrogen metabolism, including β-glucuronidase activity that may influence enterohepatic hormone recycling^38^. Prior studies have reported associations between *Bacteroidota* abundance and exogenous estradiol exposure^7^, including in individuals receiving hormone therapy^39^. Conversely, *Firmicutes* enrichment has been associated with lower estradiol states in prior work, consistent with patterns observed here. At finer taxonomic resolution, we observed enrichment of *Bacteroides* and *Ruminococcus gnavus* in TGW across both sites, taxa previously associated with estrogen-related metabolic pathways^7,40^. However, these interpretations remain indirect given the limitations of 16S sequencing used here. While the findings are consistent with a potential effect of FHT on the rectal microbiota, functional validation using metagenomics or metabolomics is required. Serum estradiol concentrations were therapeutic only in ATL TGW, which may explain stronger TGW-MSM differences in ATL compared with BKK, where hormone exposure was subtherapeutic.

Despite cross-sectional differences, we did not detect significant longitudinal changes in the microbiota following FHT initiation. This could reflect subtherapeutic hormone levels in the follow-up visit. In Bangkok, over-the-counter FHT regimens are common and often insufficient to reach target estradiol or testosterone suppression levels^24^. The regimens reported by our BKK TGW participants are similar to those reported in prior studies in this population, and our serum hormone results are also similar to those of prior studies. Microbial communities may also require longer exposure to stable endocrine changes before measurable shifts occur. In addition, 16S resolution may have limited detection of subtle functional changes. Prior metagenomic work in TGW has demonstrated modest microbiota shifts after longer durations of therapeutic FHT^41,42^, suggesting that both dose and duration are critical.

Prior studies in MSM populations have reported enrichment of *Prevotellaceae* over *Bacteroidaceae*^6,18–21^, particularly in Western cohorts. In our study, *Prevotellaceae* enrichment was observed only in ATL MSM, while *Bacteroidaceae* predominance was seen in TGW at both sites and to a lesser extent in MSM in BKK. These findings suggest that *Prevotella-Bacteroides* patterns are not universal and may be shaped by geography, gender category, and behavioral context rather than sexual practice alone.

The populations in Atlanta and Bangkok, and across gender identities, represent complex phenotypes that cannot be defined by a single variable, such as hormone use. Behavioral differences, including PrEP use, RAI frequency, lubricant use, douching, and drug use, vary significantly between groups. While our sensitivity analysis demonstrated that overall group differences are unlikely to be driven by these variables, it is possible that these behavioral factors make subtle contributions to some of the observed microbiota differences. For example, higher PrEP use in ATL MSM may explain enrichment of taxa like *Catenibacterium* and *Holdemanella*, which are linked to PrEP exposure^43–45^. *Escherichia-Shigella* enrichment in BKK TGW may reflect higher rates of RAI and exposure to enteric pathogens^46,47^. These overlapping behavioral factors complicate attribution of microbiota differences to any single exposure but reflect real-world biosocial complexity.

We provide one of the more detailed characterizations of the neovaginal microbiota to date. Compared with rectal samples, neovaginal communities were enriched in skin- and oxygen-associated taxa, such as *Propionibacteriales, Corynebacteriales*, and *Actinomycetales*, likely reflecting increased oxygen exposure and surgical anatomy. The majority of participants underwent penile inversion vaginoplasty, suggesting partial persistence of penile skin-associated flora. We identified enrichment of *Porphyromonas, Peptostreptococcus, Prevotella, Dialister*, and *Mobiluncus*, consistent with prior reports^48^. Several of these overlap with the BASIC taxa cluster (Bacteria Associated with Seroconversion, Inflammation, and immune Cells), which have been associated with mucosal inflammation and HIV seroconversion in other anatomic sites^35,49^. Although direct evidence for neovaginal HIV transmission is lacking, documented cases of other neovaginal STIs indicate biological plausibility for pathogen transmission^50–52^. Given established roles of both rectal and vaginal microbiota in HIV vulnerability^5^, the neovaginal microbiota may represent an additional but understudied factor in TGW that influences acquisition of HIV and/or other STIs.

Our study is limited by a modest sample size, particularly in longitudinal analyses, and by reliance on 16S sequencing, which limits functional inference. Hormone levels in BKK participants were frequently subtherapeutic, reducing the power to detect endocrine-associated effects, and the use of different clinical hormone immunoassays between ATL and BKK limited direct comparisons between these two groups despite overall high ICCs for serum estradiol and testosterone. Behavioral differences between groups further complicate causal interpretation. Our ability to evaluate the effect of neovaginal surgical technique on microbiota composition was limited by the small number of participants with sigmoid colon pull-through neovaginas and the lack of detailed data regarding sampling depth and time since surgery. Nonetheless, the multi-site design allows identification of robust patterns across geography and gender category.

## Conclusion

Using innovative statistical approaches, we identified mucosal microbiota signatures associated with gender category and geography that persist across populations. We found evidence consistent with hormone-associated microbial patterns in TGW, particularly involving estrogen-linked taxa, although longitudinal changes were not observed under subtherapeutic hormone exposure. We also provide an expanded characterization of the neovaginal microbiota, identifying taxa potentially relevant to inflammation and HIV susceptibility. Future work incorporating metagenomics, metabolomics, and longitudinal sampling under controlled hormone exposure will be essential to define functional consequences. Assessing gender identity as a complex biosocial phenotype and recognizing its unique biological and behavioral correlates will be essential for developing HIV prevention and health strategies with real-world applicability.

## Supplementary information

Transparent Peer Review file

Supplemental Information

Reporting summary

## Data Availability

The microbiome sequencing data have been deposited in the NCBI Sequence Read Archive under BioProject accession PRJNA1441097. The demographic, hormone, and behavioral data generated during this study are available in Figshare at https://doi.org/10.6084/m9.figshare.32052063.

## Code availability

The relevant metadata and analysis scripts are available on GitHub: https://github.com/mengyu-he/biosocial-microbiome-analysis.
