# Supplementary material for: Biosocial influences of gender identity and geography on mucosal microbial phenotypes
Source: Commun Med (Lond). 2026 Jul 14;6:577. doi: 10.1038/s43856-026-01780-7 (PMC13739924; doi:10.1038/s43856-026-01780-7)
Supplement: Supplementary file 1 [file 43856_2026_1780_MOESM1_ESM.pdf]

nature portfolio

Peer Review File

Biosocial Influences of Gender Identity and Geography on Mucosal Microbial Phenotypes

Corresponding Author: Dr Vanessa Van Doren

This file contains all reviewer reports in order by version, followed by all author rebuttals in order by version.

Version 0:

Reviewer comments:

Reviewer #1

(Remarks to the Author)
This study by Van Doren et al examined the rectal and neo-vagina microbiome in MSM and TGW from two cities (Atlanta and Bangkok). The paper is well-written and clearly describes and contextualizes the microbiome characteristics of these populations. The authors also attempt to longitudinally examine the microbiome before and after gender affirming hormone therapy, but these findings seem limited by sample size. Additionally, hormone levels were also collected but did not seem to strongly correlate with the microbiome. Overall this is a useful contribution to a poorly understood area (microbiome in transgender women). I have some minor comments for the authors to consider:

- The longitudinal design is a nice addition and adds to the study, though the small samples size does limit findings. In addition, there appears to be a wide range in post-hormone therapy time point. I'm not sure the distribution of samples across that 3 to 12 months range, but if there are a cluster of samples closer to the 12 month end perhaps those might show a greater difference (allowing more time for hormone therapy to affect the microbiome). I agree with the authors that sub-therapeutic dosing is another likely reason for minimal changes.

- The authors importantly collected robust behavioral/clinical data (RAI, PrEP use, drugs, alcohol, etc) which are known to affect the microbiome. It was not clear how this was incorporated into the analysis to control for these covariates?

- Page 21: Participants on synthetic estradiol were excluded from the microbiome-estrogen analysis. How many participants data remained for this analysis? As this was already a limited sample prior to exclusion it is not suprising there were no significant findings. Spearman correlations did show some significant associations, yet this was included only in supplemental data? It might be nice to include this in the actual manuscript if possible. In addition, these correlations were only performed at the family level? Why? As this study was done with 16S there is already limited information but performing analyses at the genus level at least gives more specific findings to guide future work.

- The Discussion section is quite long. The second paragraph on page 22 repeats all information about BOUTH analysis that was already described win the Methods section. I would recommend removing this entire paragraph (page 22 line 436 through page 23 line 453. In addition, the sentence on page 26 line 528 to 530 seems redundant and unnecessary for the paragraph.

Reviewer #2

(Remarks to the Author)
This manuscript reports a multi-site study of rectal and neovaginal mucosal microbiota in transgender women (TGW) and cisgender men who have sex with men (MSM) in Atlanta (USA) and Bangkok (Thailand). Using cross-sectional and longitudinal data, the authors combine 16S rRNA sequencing with complementary statistical methods (linear decomposition modeling [LDM] and the Bottom-Up Approach to Testing Hypotheses [BOUTH]) to identify microbial signatures associated with gender category, geographic location, and feminizing hormone therapy (FHT). The work offers a particularly detailed characterization of TGW mucosal microbiota and highlights geographic and biosocial influences relevant to HIV vulnerability.
This is a technically strong, carefully executed study that addresses an important gap in clinical microbiome research. Key strengths include the multi-site cohort, rigorous and transparent analytic framework, and integration of behavioral and

hormonal data, with conclusions that are generally supported by the results. The manuscript would be further improved by clarifying a few conceptual and methodological points and by strengthening transparency around data and code availability to enhance reproducibility. The application of BOUTH to identify biologically interpretable driver taxa is a notable contribution, and the neovaginal microbiota data are especially valuable given the limited existing literature.

Specific comments

1. Conceptual use of the term "gender identity"
Gender identity is operationalized analytically as a categorical comparison between TGW and MSM. However, I am concerned that gender identity does not accurately represent the differences between MSM and TGW. For example, this study only included TGW who use FHT, and therefore the TGW in this study represent a select part of the larger population of people with a transfeminine gender identity. Additionally, TGW and MSM differ in many covariates (e.g., RAI frequency, PrEP use, douching, prior STIs, substance use) that are not necessarily characteristics of their gender (sexuality of MSM is not an attribute of their gender identity). I recognize that the authors are using gender category as a pragmatic grouping variable to capture a constellation of biological and behavioral factors. While I cannot suggest a one-word tidy alternative to "gender," I do think the authors should consider adopting a more accurate term that better reflects the differences between these two groups.
As the authors acknowledge, teasing out the effects of the numerous confounding factors (behavior / biosocial) when interpreting TGW vs MSM (and site) differences if difficult. The authors note that their LDM analysis adjusting for potential confounders, but the covariates included are not – that I can find – listed in the manuscript. Adding a list of these covariates would facilitate interpretation of the LDM.

2. Comparison in serum estradiol between Bangkok and Atlanta TGW
About a quarter of the TGW in BKK were receiving synthetic estradiol and, as the authors note, this cannot be quantified with the serum assay used. This would seem to make comparisons of serum estradiol between the two populations invalid. If my reading of these results is correct, participants on synthetic estradiol should be excluded from Figure 2A and 3A and the associated statistical tests. While it is clear they were excluded from the LDM (page 21), were they also excluded from the Spearman correlations (lines 418-422)?

3. Serum testosterone concentrations and geographical region (Atlanta vs Bangkok).
As the proportion of participants on anti-androgens differs by region, it would be informative to distinguish participants who are using anti-androgens in Figure 2 (e.g., by a different shaped point on the plot). Did any participants have orchiectomy? Is this recorded as an "anti-androgen"?
It is interesting that there is a significant difference in serum testosterone between MSM in BKK and ATL. Were the serum samples collected at the same time of day in both locations? Could there be differences in sample collection, processing, or assay that might account for these differences? Do TGW in BKK have significantly higher testosterone than TGW in ATL, if anti-androgen therapy is controlled for / excluded?
In Figure 3, there are many participants who have undetectable testosterone at baseline. Were any of these participants on anti-androgens or has they undergone orchiectomy? Again, denoting which of the 13 participants in the post-FHT category were on anti-androgens would facilitate interpretation of these data. If paired analyses were performed, adding lines joining the two timepoints for one participant might help the reader better interpret this hormone data.

4. Hormone-related interpretation and functional inference
Several taxa are discussed as being associated with estrogen metabolism or hormonal effects. Given that the study relies on 16S rRNA gene sequencing, these functional interpretations are necessarily inferred from prior literature rather than directly measured. Slightly tempering the language (e.g., taxa previously associated with estrogen metabolism) would help distinguish observed taxonomic associations from inferred functional activity.

5. Consistency in statistical test nomenclature
The Methods section specifies Wilcoxon tests for alpha diversity comparisons, whereas several table legends refer to Mann-Whitney tests. As these represent equivalent nonparametric tests, this appears to be a nomenclature issue; however, standardizing terminology and ensuring that all statistical tests are explicitly described in the Methods would improve clarity and consistency.

6. Data on neovaginal biopsies
Depending on the surgical technique used (peritoneal, sigmoid, penile inversion), neovaginal canal tissue may have different origins. If the biopsy was collected from close to the introitus (2cm depth), it is very likely to originate from penile shaft skin. However, if the neovaginal biopsy was from deeper in the neovaginal canal it could be tissue that was originally peritoneum, scrotal skin, or sigmoid tissue. Can the authors please provide data on the depth of biopsy and the surgical technique used. If participants received sigmoid vaginoplasty, were their neovaginal microbiota more similar to their rectal microbiota than participants who received peritoneal or penile inversion vaginoplasty? Were rectal and neovaginal samples paired within individuals?
Adding data on the duration of time since vaginoplasty would also facilitate interpretation of these data.

7. Correspondence between reported taxa and supplementary figures
In several instances, the number of differentially abundant taxa reported in the text does not appear to correspond clearly with the number of taxa visualized in the associated supplementary figures. It is unclear whether this discrepancy reflects filtering for visualization, taxonomic collapsing, or exclusion of low-abundance taxa. Clarifying this in the figure legends or providing a complete supplementary table listing all significant taxa would enhance transparency and reproducibility.

8. Data and code availability
The current Data Availability statement indicates that microbiome data are available upon request to the corresponding author. Given the complexity of the analytical workflow, public availability of raw sequencing data and analysis code would substantially strengthen the manuscript. Deposition of sequencing reads in a public repository (e.g., NCBI SRA), together with access to relevant metadata and analysis scripts (e.g., via GitHub or Zenodo), would align the study with current data-sharing standards of the Nature Portfolio and improve reproducibility.

9. Minor points
– Claims based on Spearman correlations based on p-values that were not adjusted for FDR should be tempered throughout.
– Please add information on the controls used during sequencing (extraction blanks, PCR negatives, decontamination steps, etc.)
– Were the same covariates included in LDM also included in the BOUTH analysis, or is it an unadjusted analysis?
– line 119: how many participants were not estrogen naïve? Did the authors consider a sensitivity analysis excluding these participants?
– line 127. Is 12 weeks a typo? Should it possibly be 1-2 weeks?
– Should citations be removed from the abstract, to comply with journal formatting guidelines?
– It would be helpful if all abbreviations (e.g., TGW, MSM, FHT, RAI, PrEP, LDM, BOUTH) were defined at first mention.
– Please add a reference to the previous work referred to on lines 108-109.
– The relevant data for Figure 2A is squashed at the bottom of the plot. Could the upper segment of the y-axis be reduced to only 10% of the total height, or some other modification be made to improve visualization of the data?
– The description of Prevotella enrichment among MSM could be slightly contextualized to reflect its geographic variability.

Reviewer #3

(Remarks to the Author)
I co-reviewed this manuscript with one of the reviewers who provided the listed reports. This is part of the Communications Medicine initiative to facilitate training in peer review and to provide appropriate recognition for Early Career Researchers who co-review manuscripts.

Version 1:

Reviewer comments:

Reviewer #1

(Remarks to the Author)
All comment were carefully considered and the authors put a lot of effort into revising the analysis and manuscript. All concerns addressed.

Reviewer #2

(Remarks to the Author)
The authors have been responsive to my comments and have addressed all major concerns. My only remaining suggestion is that the manuscript should avoid over-interpreting the absence of differences by surgical technique given n=3 sigmoid pull-through samples and unavailable sampling depth/duration since surgery.

Reviewer #3

(Remarks to the Author)
I co-reviewed this manuscript with one of the reviewers who provided the listed reports. This is part of the Communications Medicine initiative to facilitate training in peer review and to provide appropriate recognition for Early Career Researchers who co-review manuscripts.

Open Access This Peer Review File is licensed under a Creative Commons Attribution 4.0 International License, which permits use, sharing, adaptation, distribution and reproduction in any medium or format, as long as you give appropriate credit to the original author(s) and the source, provide a link to the Creative Commons license, and indicate if changes were made.
In cases where reviewers are anonymous, credit should be given to 'Anonymous Referee' and the source.
The images or other third party material in this Peer Review File are included in the article's Creative Commons license, unless indicated otherwise in a credit line to the material. If material is not included in the article's Creative Commons license and your intended use is not permitted by statutory regulation or exceeds the permitted use, you will need to obtain permission directly from the copyright holder.
To view a copy of this license, visit https://creativecommons.org/licenses/by/4.0/

May 8th, 2026

Dear Communications Medicine Editorial Staff,

We are pleased to resubmit our revised manuscript, re-titled *"Biosocial Influences of Gender Identity and Geography on Mucosal Microbial Phenotypes"* for consideration in The Clinical Microbiome Collection. We confirm that the manuscript remains original, has not been published elsewhere, and is not under consideration by any other journal.

We sincerely thank the reviewers for their thoughtful and constructive feedback, which has strengthened the manuscript. In response, we have revised the manuscript to improve clarity, transparency, and accessibility. These updates include clarifying the statistical analyses and covariates used, refining figures and tables to better illustrate the relationships between hormone therapies, orchiectomy, and serum hormone concentrations, streamlining text for readability, and clarifying the operationalization of gender identity in our analyses. We have also provided additional methodological details, moderated language inferring microbial taxa functions, and deposited microbiome sequence data into a public repository to enhance reproducibility.

Our study continues to provide multi-site, cross-sectional and longitudinal insights into mucosal microbiota in cisgender men who have sex with men and transgender women, highlighting microbiota differences by gender category and geography and the potential impact of feminizing hormone therapy. We appreciate your consideration of this revised manuscript and look forward to the possibility of publication in Communications Medicine.

Vanessa Van Doren, MD
The RISE CFAR Building
Brown University Health
11 Fourth Street, Room 154
Providence, RI 02906
(401) 793-4151
vvandoren@brownhealth.org

--

Reviewer #1 (Remarks to the Author):

This study by Van Doren et al examined the rectal and neo-vagina microbiome in MSM and TGW from two cities (Atlanta and Bangkok). The paper is well-written and clearly describes and contextualizes the microbiome characteristics of these populations. The authors also attempt to longitudinally examine the microbiome before and after gender affirming hormone therapy, but these findings seem limited by sample size. Additionally, hormone levels were also collected but did not seem to strongly correlate with the microbiome. Overall this is a useful contribution to a poorly understood area (microbiome in transgender women). I have some minor comments for the authors to consider:

Comment 1: The longitudinal design is a nice addition and adds to the study, though the small samples size does limit findings. In addition, there appears to be a wide range in post-hormone therapy time point. I'm not sure the distribution of samples across that 3 to 12 months range, but if there are a cluster of samples closer to the 12-month end perhaps those might show a greater difference (allowing more time for hormone therapy to affect the microbiome). I agree with the authors that sub-therapeutic dosing is another likely reason for minimal changes.

Response 1: Thank you for this comment. For the 21 longitudinal participants who initiated estradiol as part of their FHT, the minimum time on estradiol was 90 days, and the maximum was 350. Most were in the 100-200 day range, however, and so comparing extremes would be limited given the very small sample size. Additionally, only one of the 21 participants demonstrated a goal serum estradiol concentration of >100 pg/mL after FHT initiation.

For the 15 longitudinal participants who initiated anti-androgen therapy as part of their FHT, the minimum time on anti-androgen was 91 days, and the maximum was 4,223 (two participants had orchiectomies a few years prior to the study, which skews the data – excluding these, the max was 350, and most were again in the 100-200 day range). Of these 15 participants, only six suppressed their testosterone to < 50 ng/dL, and two of these had had orchiectomies, and so there was essentially no antiandrogen intervention for those two participants in the longitudinal study.

I plotted out the change in estradiol concentration versus the days on estradiol below with a linear regression line, and there is no significant relationship between the two (slope = -0.008, p = 0.9, R2 = 0.0003):

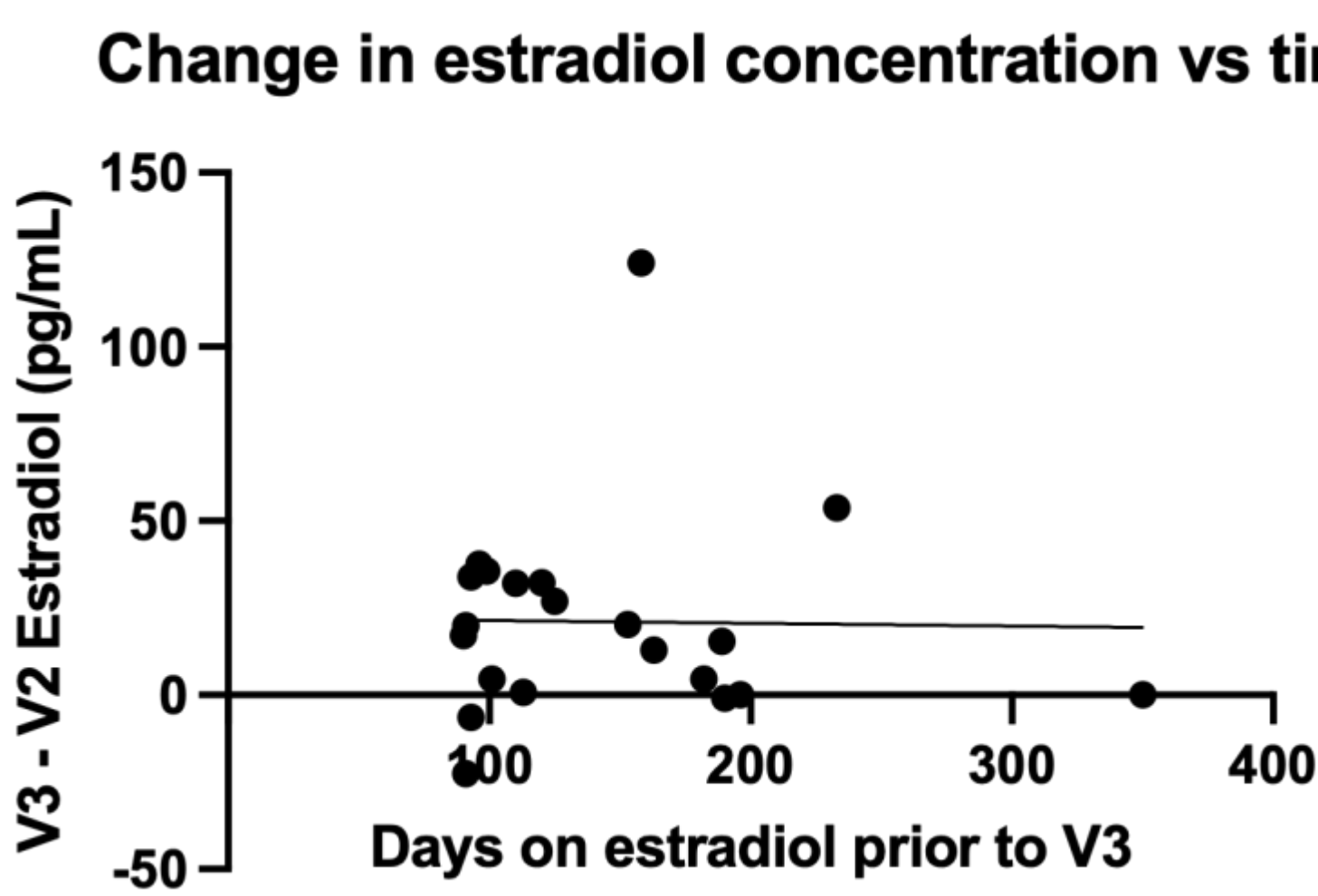

I repeated this after removing the four participants on synthetic estradiol, and while the slope becomes positive (0.15), it is still not a significant association (p = 0.4, R2 = 0.05):

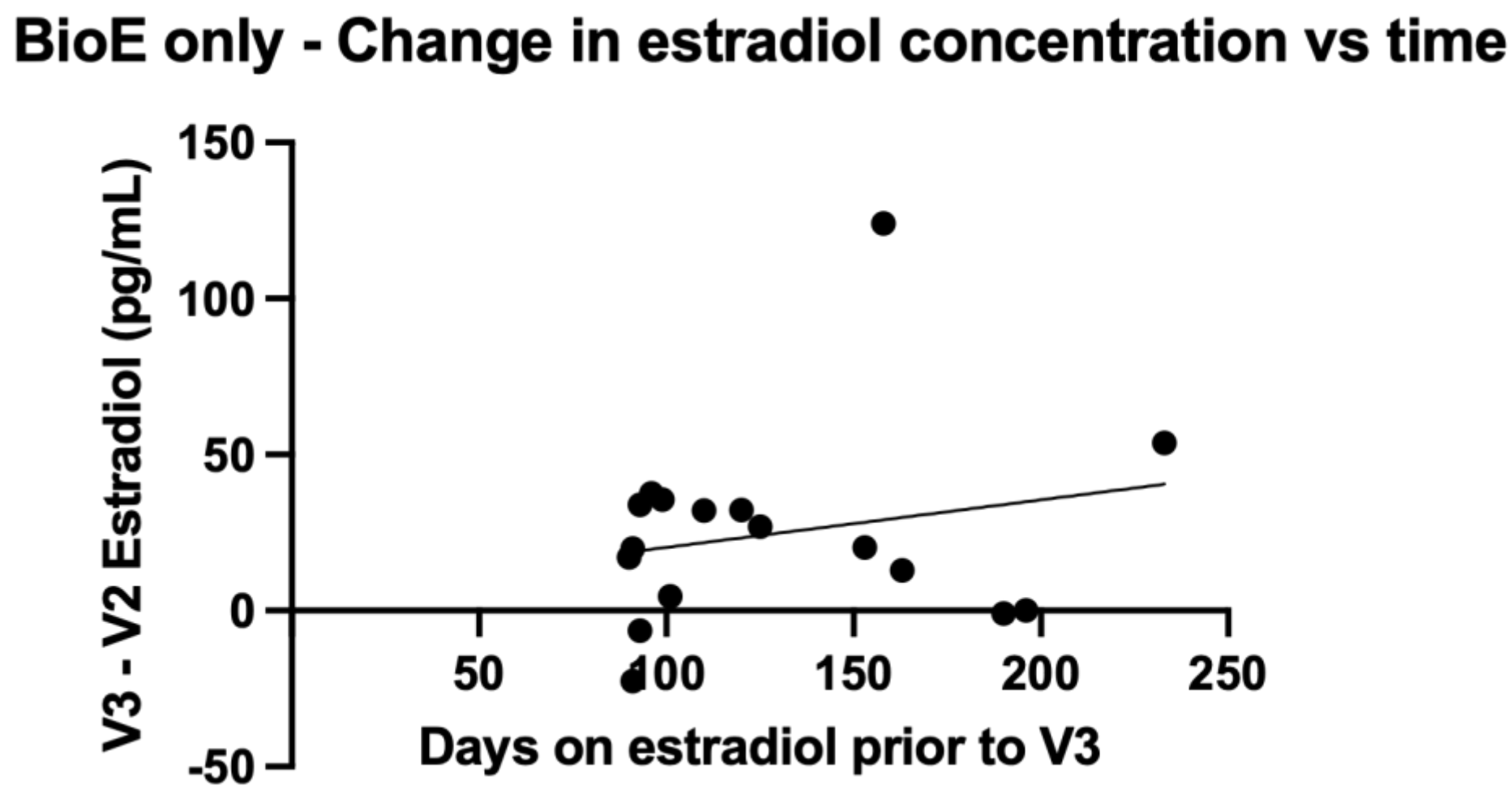

Finally, here is the same data showing the lack of relationship between anti-androgen therapy and serum testosterone changes (slope = 0.03, p = 0.8, R2 = 0.006):

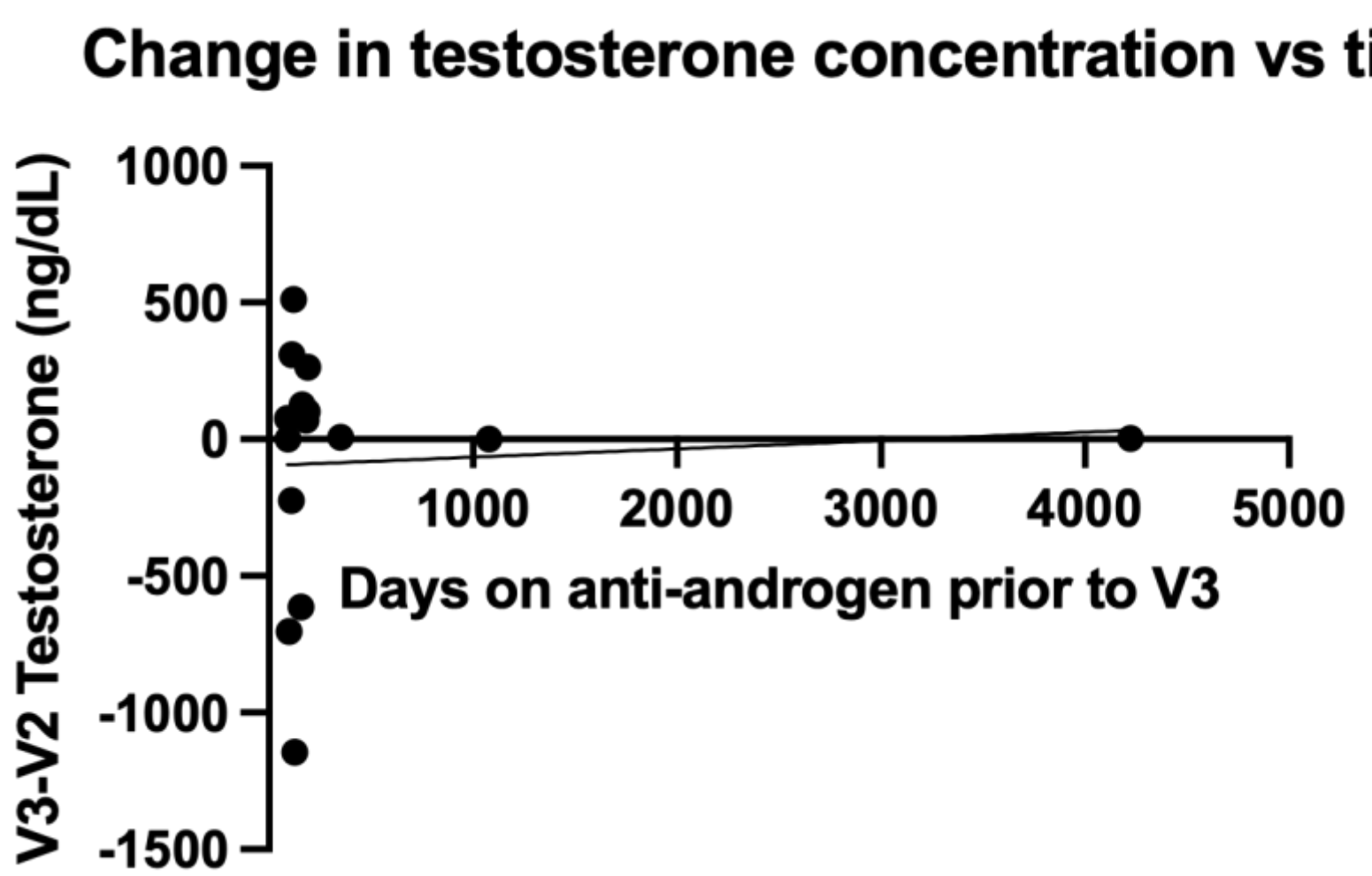

Because of the time on FHT had no effect on serum hormone concentrations, we do not expect that time on hormone would affect rectal microbiota composition either. The time on FHT also does not cluster at the extremes of the 3 month – 12 month window, and so it is not possible to do this analysis comparing these two groups.

Comment 2: The authors importantly collected robust behavioral/clinical data (RAI, PrEP use, drugs, alcohol, etc.) which are known to affect the microbiome. It was not clear how this was incorporated into the analysis to control for these covariates?

Response 2: Thank you for this important comment. In our primary analyses, we adjusted for batch and study site (when appropriate) but did not initially include additional behavioral covariates. We made this decision due to concerns about limited sample size, the potential for

reduced statistical power, and unstable parameter estimates when including multiple correlated variables.

Many of the collected behavioral factors (e.g., PrEP use, sexual practices, douching, and substance use) are not independent of one another and are also closely associated with the primary grouping variables (gender identity and study site). As shown in Table 1, these variables differ systematically between groups, suggesting that they may represent components of the broader biosocial context captured by gender identity and location, rather than functioning as simple independent confounders. As such, including them simultaneously in the primary models risks adjusting for variables that are conceptually intertwined with, or potentially on the causal pathway of, the exposures of interest.

To address the reviewer's concern and evaluate the robustness of our findings, we conducted additional sensitivity analyses in which we repeated all cross-sectional LDM and BOUTH analyses including the seven covariates that differed significantly between groups (RAI frequency, PrEP use, prior STI, douching, tobacco use, alcohol use, and drug use). As expected, inclusion of these variables resulted in attenuation of statistical significance, likely reflecting reduced power due to additional model complexity, multiple testing burden, and loss of observations from incomplete covariate data.

Importantly, however, the identified patterns remained highly consistent. Across all comparisons, 100% of named taxa that were identified as differentially abundant in the primary LDM analyses retained the same direction of effect after covariate adjustment:

ATL TGW vs MSM:

- Increased in TGW:
    - Relative abundance (RA): 8/8 (100%) concordant direction of effect
    - Presence absence (PA): 7/7 (100%) concordant direction of effect
- Increased in MSM:
    - RA: 10/10 (100%) concordant direction of effect
    - PA: 11/11 (100%) concordant direction of effect

BKK TGW vs MSM:

- Increased in TGW:
    - RA: 1/1 (100%) concordant direction of effect
    - PA: 3/3 (100%) concordant direction of effect
- Increased in MSM:
    - RA: 2/2 (100%) concordant direction of effect
    - PA: 1/1 (100%) concordant direction of effect

TGW: BKK vs ATL:

- Increased in BKK:
    - RA: 4/4 (100%) concordant direction of effect
    - PA: 5/5 (100%) concordant direction of effect

- Increased in ATL:
    - RA: 16/16 (100%) concordant direction of effect
    - PA: 3/3 (100%) concordant direction of effect

MSM: BKK vs ATL:
- Increased in BKK:
    - RA: 8/8 (100%) concordant direction of effect
    - PA: 42/42 (100%) concordant direction of effect
- Increased in ATL:
    - RA: 45/45 (100%) concordant direction of effect
    - PA: 14/14 (100%) concordant direction of effect

Similarly in the BOUTH analysis, fewer signals were detected, but the signals that remained were generally consistent with the main findings. Some taxa previously identified as drivers at the phylum level were represented by drivers at lower taxonomic levels after inclusion of covariates.

Together, these findings suggest that while statistical significance is sensitive to model specification and sample size, the overall structure and directionality of the observed microbiome differences are robust to the adjustment of additional covariates. We have added text to the Methods (Marked Up Version: lines 599-608), Results (Marked Up Version: lines 1032-1039), and Discussion (Marked Up Version: lines 1613-1615) to clarify our modeling approach and to present these sensitivity analyses.

Comment 3: Page 21: Participants on synthetic estradiol were excluded from the microbiome-estrogen analysis. How many participants data remained for this analysis? As this was already a limited sample prior to exclusion it is not surprising there were no significant findings. Spearman correlations did show some significant associations, yet this was included only in supplemental data? It might be nice to include this in the actual manuscript if possible. In addition, these correlations were only performed at the family level? Why? As this study was done with 16S there is already limited information but performing analyses at the genus level at least gives more specific findings to guide future work.

Response 3: We conducted serum estradiol-rectal microbiota relative abundance analyses on 100 participants total (all cross-sectional TGW; 75 from BKK and 25 from ATL). There were 97 participants in the cross-sectional BKK TGW group, and 22 were removed because they were taking synthetic estradiol, which is not detected by clinical serum estradiol assays. 75 participants in the cross-sectional BKK TGW group remained for this analysis. None of the 25 ATL TGW were taking synthetic estradiol, and so they were all included in this analysis. We edited the paragraph (Marked Up Version: lines 1126-1128) to describe the sample sizes more clearly.

We initially conducted our serum estradiol– and testosterone-microbiota relative abundance LDMs in this 100-participant subset at the genus level. Neither LDM showed significant global associations, and none of the individual taxa were significantly different in either LDM after adjusting for multiple comparisons. To avoid conducting an exploratory "fishing expedition" by running LDMs for both estradiol and testosterone across all phylogenetic levels, we decided to first conduct Spearman correlations for serum estradiol and testosterone at all available phylogenetic levels (phyla, class, order, family). Based on these results, we selected the most appropriate phylogenetic level for subsequent, more rigorous LDM analyses for both hormones.

<u>We have summarized the serum estradiol – microbiota relative abundance Spearman correlation data at each phylogenetic level as follows:</u>
Phyla: 9 phyla evaluated, 0 had a significant (unadjusted p<0.05) association with serum estradiol
Class: 2/11 (18.2%; Bacteroidia, p=0.008, r=0.19; and Campylobacteria, p=0.003, r=0.21) had significant associations with serum estradiol
Order: 3/25 (12%; Bacteroidales, p=0.008, r=0.19; Campylobacterales, p=0.003, r=0.21; Veillonellales-Selenomonadales, p=0.005, r=-0.2) had significant associations with serum estradiol
Family: 6/39 (15%.4; Bacteroidaceae, p=0.03, r=0.15; Campylobacteraceae, p=0.003, r=0.21; Enterobacteriaceae, p=0.4, r=-0.14; Pasteurellaceae, p=0.008, r=-0.19; Porphyromonadaceae, p=0.001, r=0.23; Selenomonadaceae, p=0.0003, r=-0.25) had significant associations with serum estradiol

<u>For serum testosterone:</u>
Phyla: 9 phyla evaluated, 0 had a significant (unadjusted p<0.05) association with serum testosterone
Class: 0/11 had a significant association with serum testosterone
Order: 1/25 (4%; Monoglobales, p=0.007, r=0.19) had a significant association with serum testosterone
Family: 5/39 (12.8%; Bacteroidaceae, p=0.02, r=-0.16; Lactobacillaceae, p=0.005, r=-0.2; Monoglobaceae, p=0.007, r=-0.2; Porphyromonadaceae, p=0.03, r=-0.15; Rikonellaceae, p=0.004, r=-0.2) had a significant association with serum testosterone

Family-level analyses exhibited the largest number of unadjusted significant associations across both estradiol and testosterone. Consequently, we repeated the LDM analyses at the family level for both hormones. These LDMs did not reveal significant associations between serum hormone levels and microbiota relative abundance.

We opted to publish the unadjusted Spearman correlations as exploratory data to support hypothesis generation while avoiding overemphasis on their statistical significance. Accordingly, these results were included in the supplemental tables, which allows interested researchers to access them without presenting them as primary findings. Given that the main manuscript already contains nine figures presenting more rigorous analyses, we determined that the supplemental tables were the most appropriate location for the exploratory data.

Comment 4: The Discussion section is quite long. The second paragraph on page 22 repeats all information about BOUTH analysis that was already described win the Methods section. I would recommend removing this entire paragraph (page 22 line 436 through page 23 line 453. In addition, the sentence on page 26 line 528 to 530 seems redundant and unnecessary for the paragraph.

Response 4: Thank you – we removed most of the BOUTH paragraph from the Discussion, and we removed the sentence on page 26 as recommended. We also conducted broad extensive edits throughout the discussion and parts of the methods to produce a more streamlined manuscript.

Reviewer #2 (Remarks to the Author):

This manuscript reports a multi-site study of rectal and neovaginal mucosal microbiota in transgender women (TGW) and cisgender men who have sex with men (MSM) in Atlanta (USA) and Bangkok (Thailand). Using cross-sectional and longitudinal data, the authors combine 16S rRNA sequencing with complementary statistical methods (linear decomposition modeling [LDM] and the Bottom-Up Approach to Testing Hypotheses [BOUTH]) to identify microbial signatures associated with gender category, geographic location, and feminizing hormone therapy (FHT). The work offers a particularly detailed characterization of TGW mucosal microbiota and highlights geographic and biosocial influences relevant to HIV vulnerability. This is a technically strong, carefully executed study that addresses an important gap in clinical microbiome research. Key strengths include the multi-site cohort, rigorous and transparent analytic framework, and integration of behavioral and hormonal data, with conclusions that are generally supported by the results. The manuscript would be further improved by clarifying a few conceptual and methodological points and by strengthening transparency around data and code availability to enhance reproducibility. The application of BOUTH to identify biologically interpretable driver taxa is a notable contribution, and the neovaginal microbiota data are especially valuable given the limited existing literature.

Specific comments:

Comment 1: Conceptual use of the term "gender identity"

Gender identity is operationalized analytically as a categorical comparison between TGW and MSM. However, I am concerned that gender identity does not accurately represent the differences between MSM and TGW. For example, this study only included TGW who use FHT, and therefore the TGW in this study represent a select part of the larger population of people with a transfeminine gender identity. Additionally, TGW and MSM differ in many covariates (e.g., RAI frequency, PrEP use, douching, prior STIs, substance use) that are not necessarily characteristics of their gender (sexuality of MSM is not an attribute of their gender identity). I recognize that the authors are using gender category as a pragmatic grouping variable to capture a constellation of biological and behavioral factors. While I cannot suggest a one-word

tidy alternative to “gender,” I do think the authors should consider adopting a more accurate term that better reflects the differences between these two groups.

As the authors acknowledge, teasing out the effects of the numerous confounding factors (behavior / biosocial) when interpreting TGW vs MSM (and site) differences if difficult. The authors note that their LDM analysis adjusting for potential confounders, but the covariates included are not – that I can find – listed in the manuscript. Adding a list of these covariates would facilitate interpretation of the LDM.

Response 1: Thank you for this thoughtful comment. We agree that the analytic comparison between TGW and MSM reflects a constellation of biological, hormonal, behavioral, and social factors rather than gender identity alone.

In our study, TGW participants were individuals receiving FHT and therefore represent a hormonally defined subset of the broader transfeminine population. We agree that differences observed between TGW and MSM reflect multiple biosocial influences, including hormone exposure, sexual practices, and other behavioral or clinical factors, rather than gender identity per se.

To address this concern, we have revised the manuscript to clarify that TGW and MSM are used as participant categories defined by self-reported gender identity and study eligibility criteria rather than as a direct proxy for gender identity alone. We kept the "gender identity" language in the parts of the manuscript simply stating the self-reported gender identities of participants in the study groups or describing the concept generally, but in the sections associating those groups with outcomes (specifically, the title and left column of Table 4, the title of Figures 4 and 5, and throughout the text) we changed “gender identity” to “gender category” to make it clear that the gender identity isn’t necessarily driving the association, and that there are many other variables that differ between these gender categories, which were created for this study, that may be contributing to the association. We also now explicitly note in the Methods --> Clinical Cohort section (Marked Up Version: lines 143-154) that TGW participants in this study were receiving FHT and therefore represent a specific subgroup within the transfeminine population. We also added a paragraph in the Discussion (Marked Up Version: lines 1182-1185) explaining that observed microbiome differences between TGW and MSM likely reflect a combination of biological and behavioral factors associated with these groups.

Reviewer #1 also raised the question about whether and which covariates were included in the models – please see our response to Reviewer #1, Comment 2 above.

Comment 2: Comparison in serum estradiol between Bangkok and Atlanta TGW
About a quarter of the TGW in BKK were receiving synthetic estradiol and, as the authors note, this cannot be quantified with the serum assay used. This would seem to make comparisons of serum estradiol between the two populations invalid. If my reading of these results is correct, participants on synthetic estradiol should be excluded from Figure 2A and 3A and the

associated statistical tests. While it is clear they were excluded from the LDM (page 21), were they also excluded from the Spearman correlations (lines 418-422)?

Response 2: Yes –participants taking synthetic estradiol were excluded from the Spearman correlation analyses evaluating associations between serum estradiol concentration and microbiota relative abundance, in addition to being excluded from the more rigorous LDM analyses. We have added a clarifying clause in this section to clarify this.

However, these participants were not excluded from the descriptive Figures 2A and 3A. The purpose of these figures is to illustrate the variability in serum estradiol concentrations that we were able to measure across participants, rather than to test associations with microbiota or other variables. Excluding participants from these descriptive figures could obscure the true range of measured hormone concentrations in the population. In contrast, for analyses specifically assessing associations between serum estradiol and microbiota, it was necessary to exclude participants with inaccurate or unmeasurable serum estradiol values. Including them in the descriptive figures while excluding them from the correlation and LDM analyses allows us to present the full context of hormone measurement variability while maintaining rigor in hypothesis-driven analyses.

While reviewing figure 2 for this resubmission, we observed a greater frequency of high estradiol values in both BKK cohorts compared to ATL cohorts. As described in the Methods, serum hormones were measured using different clinical immunoassays at the two sites. To assess comparability, we previously calculated intraclass correlation coefficients (ICCs) using a subset of 41 samples and found high agreement for estradiol (0.86) and testosterone (0.92).

However, upon further evaluation and consultation with our statisticians, we recognized that ICC estimates may not adequately reflect agreement across the full range of values, particularly at the extremes. Given the observed distributional differences and the use of different assays, direct comparison of individual hormone concentrations between ATL and BKK participants may therefore be inappropriate.

In light of this, we have revised Figure 2 to present the proportion of TGW in ATL and BKK who demonstrated therapeutic serum estradiol and testosterone concentrations, as defined by Endocrine Society Guidelines. This approach better aligns with the clinical objective of the analysis, which is to assess the extent to which participant demonstrated target hormone ranges, while avoiding potentially misleading cross-assay comparisons of absolute values.

We have retained the original figure as a supplemental figure for completeness and transparency.

Figure 3 remains unchanged, as it presents longitudinal hormone concentrations within the BKK TGW cohort, all measured using the same assay across participants and timepoints, and is therefore not subject to the same comparability concerns.

Comment 3: Serum testosterone concentrations and geographical region (Atlanta vs Bangkok) As the proportion of participants on anti-androgens differs by region, it would be informative to distinguish participants who are using anti-androgens in Figure 2 (e.g., by a different shaped point on the plot). Did any participants have orchiectomy? Is this recorded as an "anti-androgen"?

It is interesting that there is a significant difference in serum testosterone between MSM in BKK and ATL. Were the serum samples collected at the same time of day in both locations? Could there be differences in sample collection, processing, or assay that might account for these differences? Do TGW in BKK have significantly higher testosterone than TGW in ATL, if anti-androgen therapy is controlled for / excluded?

In Figure 3, there are many participants who have undetectable testosterone at baseline. Were any of these participants on anti-androgens or has they undergone orchiectomy? Again, denoting which of the 13 participants in the post-FHT category were on anti-androgens would facilitate interpretation of these data. If paired analyses were performed, adding lines joining the two timepoints for one participant might help the reader better interpret this hormone data.

Response 3: Thank you for this feedback. In Figures 2B (cross-sectional testosterone) and 3B (longitudinal testosterone), we changed the circles to triangles for the 18/25 ATL TGW and 65/97 BKK TGW (Figure 2) who were on anti-androgen therapy and the 15/21 BKK TGW (Figure 3) who started anti-androgen therapy as part of the FHT regimen initiated during the study. Two of the longitudinal BKK TGW had undergone orchiectomy, and so we updated Figure 3B to reflect this and changed these two triangles to red in both the pre-FHT and post-FHT plots (since they were effectively on anti-androgen therapy throughout the study and no change from a minimal baseline should be expected – they did have undetectable/near-undetectable serum testosterone at both timepoints as expected). Two additional longitudinal participants did not report being on anti-androgen therapy at the beginning of the study and did not report orchiectomy but had near-undetectable testosterone concentrations at the pre-FHT and post-FHT timepoints. Because this is self-reported data, it is possible that these two participants could have been on anti-androgen therapy in the six months prior to study enrollment.
None of the cross-sectional ATL TGW had undergone orchiectomy. Two of the cross-sectional BKK TGW had undergone orchiectomy, and so we updated Figure 2, and the corresponding Table 2, to reflect this.

For the cross-sectional study, we did an additional analysis as suggested separating the data from TGW into these four groups: 18/25 ATL TGW on anti-androgen therapy, 7/25 ATL TGW not on anti-androgen therapy, the 65/97 BKK TGW on anti-androgen therapy, and 32/97 BKK TGW not on anti-androgen therapy. Kruskal-Wallis comparison of the groups did not show a significant difference ($p=0.16$). The relationship between anti-androgen status and serum testosterone concentration in these cross-sectional groups is also now more apparent from the suggested changes to supplemental Figure 1 described above.

We appreciate the reviewer's question regarding the timing of serum hormone collection at the two sites. Unfortunately, the exact time of day at which these assays were obtained was not routinely collected at both sites.

Comment 4: Hormone-related interpretation and functional inference
Several taxa are discussed as being associated with estrogen metabolism or hormonal effects. Given that the study relies on 16S rRNA gene sequencing, these functional interpretations are necessarily inferred from prior literature rather than directly measured. Slightly tempering the language (e.g., taxa previously associated with estrogen metabolism) would help distinguish observed taxonomic associations from inferred functional activity.

Response 4: Thank you for this feedback. We updated the "Gender category and hormone-associated signatures" paragraph in the Discussion to make it clear that 16S limits our ability to directly assess function in this study and that all the microbiome-estrogen associations we discuss are based on prior literature.

Comment 5: Consistency in statistical test nomenclature
The Methods section specifies Wilcoxon tests for alpha diversity comparisons, whereas several table legends refer to Mann-Whitney tests. As these represent equivalent nonparametric tests, this appears to be a nomenclature issue; however, standardizing terminology and ensuring that all statistical tests are explicitly described in the Methods would improve clarity and consistency.

Response 5: We thank the reviewer for this comment and agree that these represent the same statistical approach. To improve clarity and consistency, we have revised the manuscript text, figure legends, and Supplemental Figure 7 to refer to these analyses uniformly as "Wilcoxon tests." We also added a section in the Methods to explicitly describe the more basic statistical analyses conducted for Tables 2 and 3 and Figures 2 and 3, above the microbiota statistical analysis section.

Comment 6: Data on neovaginal biopsies
Depending on the surgical technique used (peritoneal, sigmoid, penile inversion), neovaginal canal tissue may have different origins. If the biopsy was collected from close to the introitus (2cm depth), it is very likely to originate from penile shaft skin. However, if the neovaginal biopsy was from deeper in the neovaginal canal it could be tissue that was originally peritoneum, scrotal skin, or sigmoid tissue. Can the authors please provide data on the depth of biopsy and the surgical technique used. If participants received sigmoid vaginoplasty, were their neovaginal microbiota more similar to their rectal microbiota than participants who received peritoneal or penile inversion vaginoplasty? Were rectal and neovaginal samples paired within

individuals? Adding data on the duration of time since vaginoplasty would also facilitate interpretation of these data.

Response 6: Thank you for this insightful comment. All of the neovaginal sampling was done by swab collection, not biopsy, from the neovaginal vault at the Bangkok site. The Bangkok study site was conducted through collaborators affiliated with the U.S. Centers for Disease Control and Prevention (CDC). The original datasets that we received from the Bangkok site did not include the depth of neovaginal swab collection. Following completion of data collection and transfer of the analytic dataset, changes in institutional policies affecting CDC personnel resulted in the withdrawal of these collaborators from participating in ongoing study activities, including data clarification and manuscript preparation. As a result, we are unable to obtain additional site-level details that are not contained within the archived dataset.

Rectal and neovaginal samples were not paired within individuals due to the limited sample size for neovaginal sampling. We did assess whether neovaginal microbiota composition differed by source tissue type. Of the 13 participants with neovaginal sampling, 12 had known surgical technique. Of these, nine had penile inversion and three had sigmoid colon pull through. No significant differences in microbiota composition were observed between surgical types. Additionally, the three samples from the colon pull through tissue did not cluster with the 97 rectal samples, although this assessment is extremely limited by sample size:

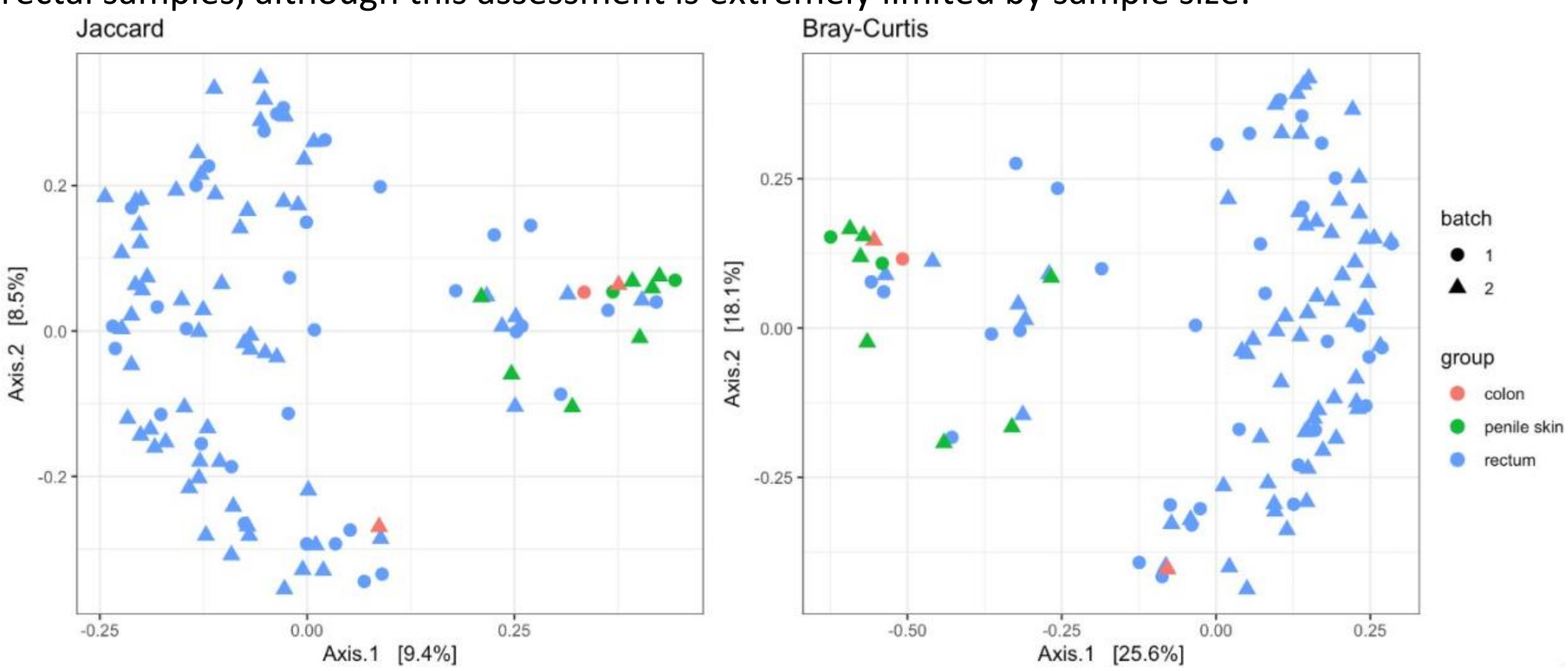

Comment 7: Correspondence between reported taxa and supplementary figures
In several instances, the number of differentially abundant taxa reported in the text does not appear to correspond clearly with the number of taxa visualized in the associated supplementary figures. It is unclear whether this discrepancy reflects filtering for visualization, taxonomic collapsing, or exclusion of low-abundance taxa. Clarifying this in the figure legends or providing a complete supplementary table listing all significant taxa would enhance transparency and reproducibility.

Response 7: Thank you for this comment. Unidentified and uncultured taxa were not included in these supplemental figures because they did not add any useful information and made the figures more cluttered and difficult to read. For example, Supplemental Figure 1 lists 21 differentially abundant taxa but only shows 18 in the figure because three were unidentified/uncultured, and it lists 19 taxa with differential probability of presence but only shows 18 taxa with differential probability of presence in the figure because one was unidentified/uncultured. At the end of these supplemental figure legends, we stated "Unidentified and uncultured taxa are not included in this figure" for transparency about this discrepancy.

Comment 8: Data and code availability
The current Data Availability statement indicates that microbiome data are available upon request to the corresponding author. Given the complexity of the analytical workflow, public availability of raw sequencing data and analysis code would substantially strengthen the manuscript. Deposition of sequencing reads in a public repository (e.g., NCBI SRA), together with access to relevant metadata and analysis scripts (e.g., via GitHub or Zenodo), would align the study with current data-sharing standards of the Nature Portfolio and improve reproducibility.

Response 8: Thank you for this comment. We have uploaded the microbiome data into NCBI SRA (SUB16106263, BioProject PRJNA1441097). Here is a reviewer link to this data: https://dataview.ncbi.nlm.nih.gov/object/PRJNA1441097?reviewer=2q6nb6b87lrhe86v6dekn049ce

We have uploaded the relevant metadata and analysis scripts to GitHub. Here is the link: https://github.com/mengyu-he/biosocial-microbiome-analysis

We have also uploaded the demographic, hormone, and behavioral data to FigShare – here is the link: https://figshare.com/s/21f0238a6e2b453c7cc0

Finally, we updated the Data Availability Statement to include these references.

Comment 9: Minor points
A. Claims based on Spearman correlations based on p-values that were not adjusted for FDR should be tempered throughout.

Response 9A: Thank you for this comment. The Spearman correlation analyses were intended as exploratory/descriptive analyses to examine potential relationships among variables. We agree that because multiple correlations were evaluated and the resulting p-values were not adjusted for FDR, these results should not be interpreted as confirmatory evidence.

To address this concern, we have clarified this point in the Results section (Marked Up Version: lines 1111-1113) to explicitly state that the exploratory analyses were not adjusted for multiple comparisons and that this was a nominal significance threshold. We also carefully reviewed the Discussion section and confirmed that no conclusions or primary interpretations in the manuscript rely on these correlations. No substantive claims in the discussion are based on these analyses. These revisions are intended to ensure that readers appropriately interpret the correlation analyses as exploratory and hypothesis-generating rather than confirmatory.

B. Please add information on the controls used during sequencing (extraction blanks, PCR negatives, decontamination steps, etc.)

Response 9B: Thank you for this helpful suggestion. We have revised the Method to include additional details regarding contamination monitoring and laboratory controls used during microbiome sequencing (Marked Up Version: lines 464-474). Specifically, we now describe the inclusion of extraction blank controls and PCR negative controls, which were preprocessed alongside study samples through DNA extraction, library preparation, and sequencing to monitor potential contamination. We also added text describing standard laboratory practices used to minimize contamination during sample processing.

C. Were the same covariates included in LDM also included in the BOUTH analysis, or is it an unadjusted analysis?

Response 9C: The same covariates (batch and location) were included in the LDM and BOUTH analysis. The sensitivity analyses detailed under Reviewer 1, Comment 2 included an additional 7 covariates: RAI frequency, PrEP use, prior STI, douching, tobacco use, alcohol use, and drug use.

D. line 119: how many participants were not estrogen naïve? Did the authors consider a sensitivity analysis excluding these participants?

Response 9D: We unfortunately did not differentiate between estrogen-naïve patients and those who had been off estrogen for at least six months during data collection – this is only available in our dataset as inclusion/exclusion criteria.

E. Line 127. Is 12 weeks a typo? Should it possibly be 1-2 weeks?

Response 9E: Thank you for this comment. The up-to-12-week interval between the screening visit and the in-person visit is not a typo. The window is intentionally set to allow flexibility for participants to schedule the visit according to their availability. This approach has been consistently used in our prior studies and is standard practice in our protocol.

F. Should citations be removed from the abstract, to comply with journal formatting guidelines?
Response 9F: Thank you for this comment. The initial submission did not require journal-specific formatting, however we are now at the stage where we will reformat the submission to comply with journal formatting rules, including removing citations from the abstract.

G. It would be helpful if all abbreviations (e.g., TGW, MSM, FHT, RAI, PrEP, LDM, BOUTH) were defined at first mention.

Response 9G: Thank you for this comment. We have reviewed the manuscript to ensure that abbreviations are defined at first mention in the main body of manuscript (in addition to in the abstract).

H. Please add a reference to the previous work referred to on lines 108-109.

Response 9H: We thank the reviewer for this suggestion. The immune cell subset and HIV challenge analyses referenced in lines 108-109 (Marked Up Version: lines 169-170) are being prepared as a companion manuscript. Because this work has not yet been submitted for publication, we have edited the wording of this statement to explain that this manuscript is in preparation and not yet accepted.

I. The relevant data for Figure 2A is squashed at the bottom of the plot. Could the upper segment of the y-axis be reduced to only 10% of the total height, or some other modification be made to improve visualization of the data?

Response 9I: Thank you for this suggestion – we redid the plot with the top Y-axis segment at 10% and the bottom segment at 90%, and the data is much more readable now.

J. The description of Prevotella enrichment among MSM could be slightly contextualized to reflect its geographic variability.

Response 9J: Thank you for this suggestion. To provide additional context, we have updated this paragraph in the Discussion (Marked Up Version: lines 1580-1585) noting that the prevalence and magnitude of *Prevotella* enrichment among MSM can vary across cohorts and may be influenced by geography and other population-level factors.

<u>Reviewer #2 Requests:</u>

Request 1: The authors have been responsive to my comments and have addressed all major concerns. My only remaining suggestion is that the manuscript should avoid over-interpreting the absence of differences by surgical technique given n=3 sigmoid pull-through samples and unavailable sampling depth/duration since surgery.

Response 1: Thank you for this feedback. We updated the relevant section of the results (lines 477-480 in the tracked changes version) as follows:

"Among participants with known neovaginal surgical technique (n=12), nine had penile inversion and three had sigmoid colon pull-through. No significant differences in microbiota composition were observed between surgical types (LDM global p=0.3), although the small number of sigmoid colon pull-through samples limited statistical power for this comparison."

And added the below section to the limitations paragraph in the discussion (lines 604-607 in the track changes version) as follows:

"Our ability to evaluate the effect of neovaginal surgical technique on microbiota composition was limited by the small number of participants with sigmoid colon pull-through neovaginas and the lack of detailed data regarding sampling depth and time since surgery."
