# Supplementary material for: Biosocial influences of gender identity and geography on mucosal microbial phenotypes
Source: Commun Med (Lond). 2026 Jul 14;6:577. doi: 10.1038/s43856-026-01780-7 (PMC13739924; doi:10.1038/s43856-026-01780-7)
Supplement: Supplementary file 2 [file 43856_2026_1780_MOESM2_ESM.pdf]

Supplemental Figures

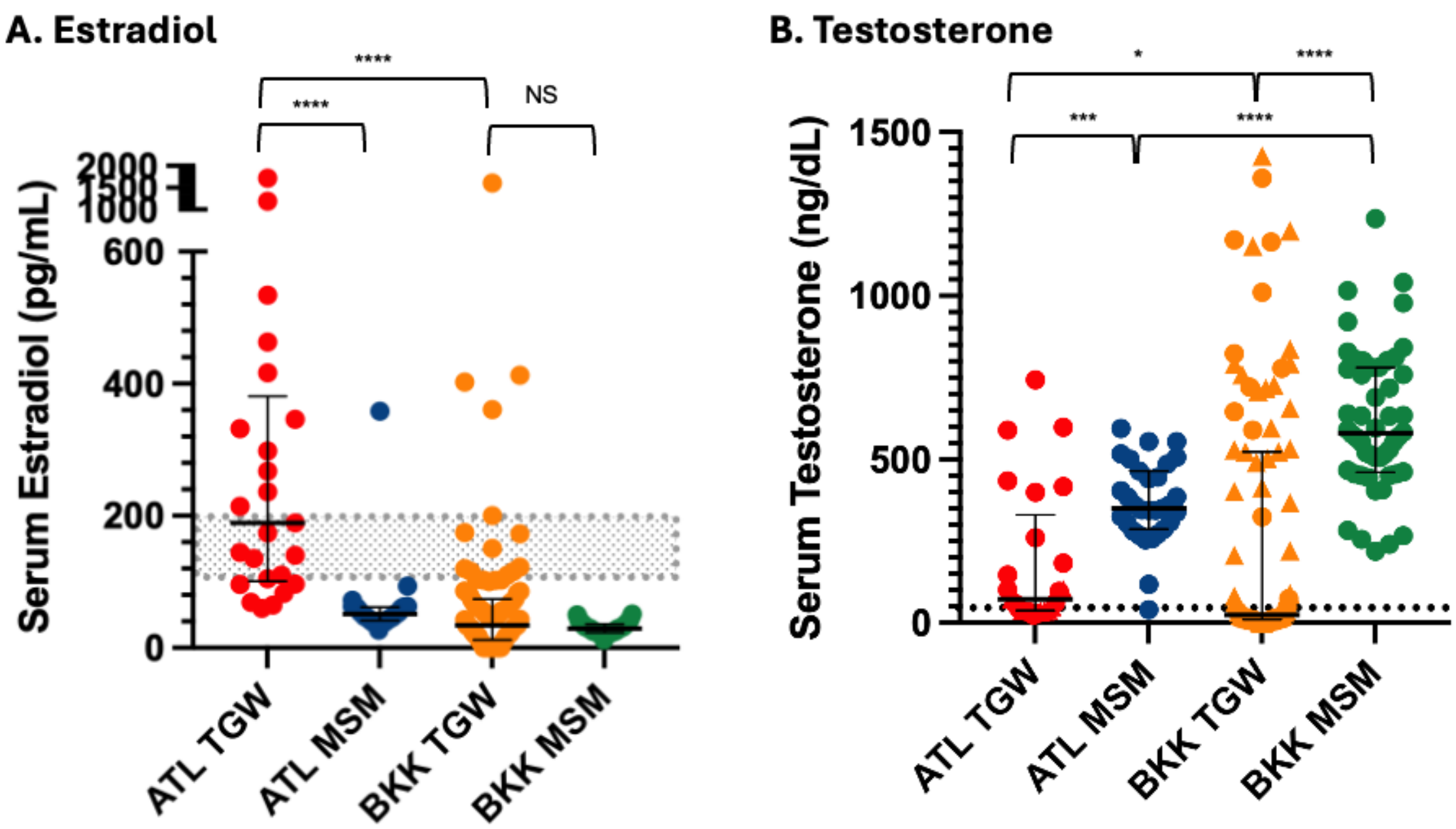

Supplemental Figure 1: Cross-Sectional Serum Hormone Concentrations. These plots display serum estradiol (A) and testosterone (B) concentration medians and interquartile ranges between the four cross-sectional study groups: ATL TGW (n=25); ATL MSM (n=33); BKK TGW (n=97); BKK MSM (n=50). 18/25 ATL TGW and 65/97 BKK TGW were taking anti-androgen therapy as part of their FHT; these participants are indicated with triangles. The shaded area (estradiol) and dotted line (testosterone) indicate therapeutic concentrations for TGW per Endocrine Society Guidelines (100-200 pg/mL for serum estradiol and < 50 ng/dL for serum testosterone). Comparisons were conducted with two-sided Wilcoxon tests (A. Estradiol: ATL TGW vs ATL MSM p = <0.0001; ATL TGW vs BKK TGW p = <0.0001; BKK TGW vs BKK MSM p = 0.15; B. Testosterone: ATL TGW vs ATL MSM p = 0.0002; ATL TGW vs BKK TGW p = 0.03; ATL MSM vs BKK MSM p = <0.0001; BKK TGW vs BKK MSM p = <0.0001). * = p < 0.05, ** = p < 0.01, *** = p < 0.001, **** = p < 0.0001.

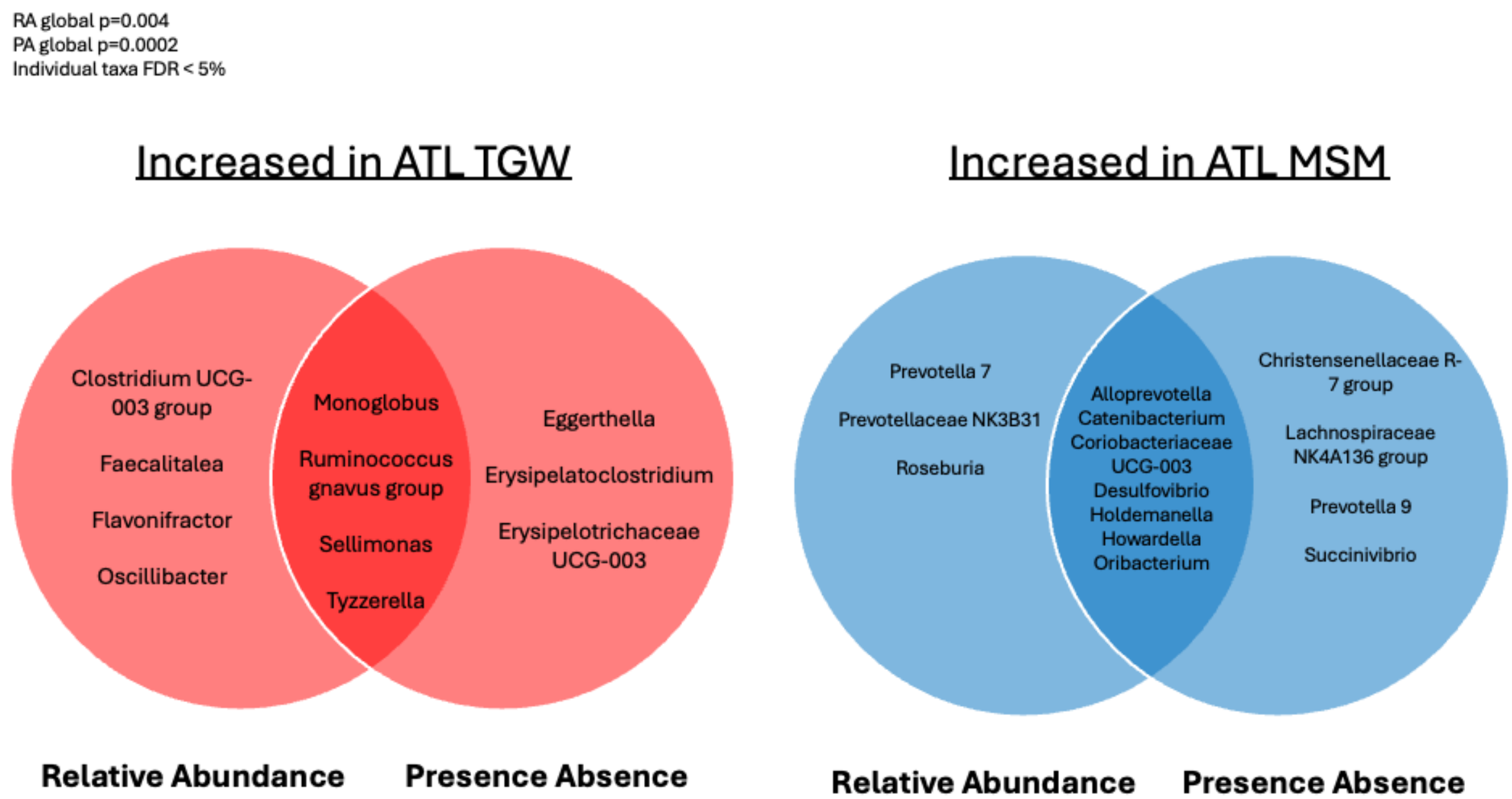

Supplemental Figure 2: Gender Identity Comparisons in Atlanta by LDM. The rectal microbiota significantly differed between ATL TGW (transgender women in Atlanta; n=25) and ATL MSM (cisgender men who have sex with men in Atlanta; n=33) by relative abundance linear decomposition modeling (LDM; global p=0.004; 21 differentially-abundant taxa) and presence-absence LDM (global p=0.0002; 19 taxa with differential probability of presence). Alpha diversity was not significantly different (p=0.12). Unidentified and uncultured taxa are not included in this figure.

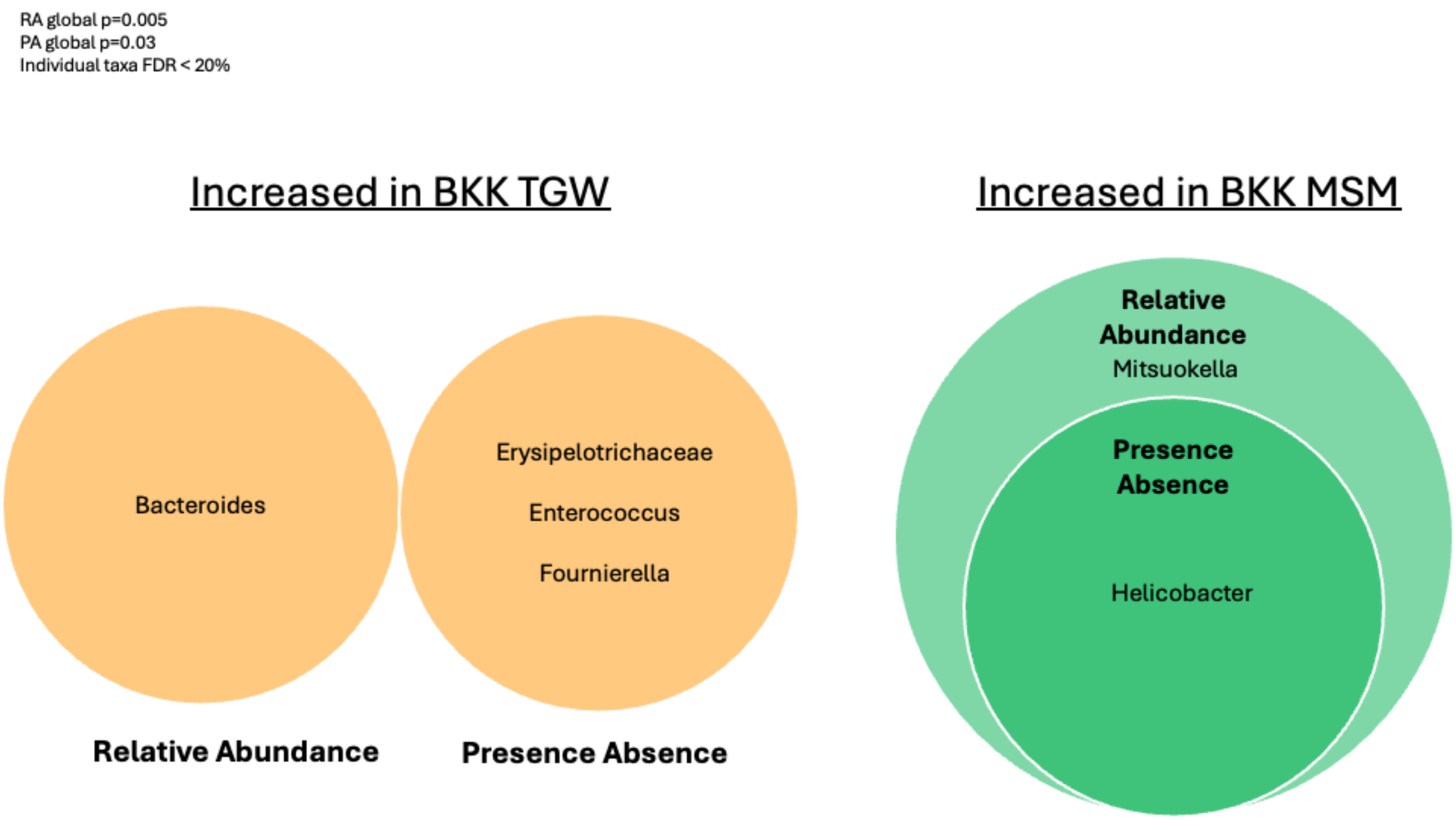

Supplemental Figure 3: Gender Identity Comparisons in Bangkok by LDM. The rectal microbiota significantly differed between BKK TGW (transgender women in Bangkok; n=97) and BKK MSM (cisgender men who have sex with men in Bangkok; n=50) by relative abundance linear decomposition modeling (LDM; global p=0.005; 0 differentially-abundant taxa at FDR<5% and 3 differentially-abundant taxa with FDR<20%) and presence-absence LDM (global p=0.03; 0 differentially-abundant taxa at FDR<5% and 4 differentially-abundant taxa with FDR<20%). Alpha diversity was not significantly different (p=0.96).

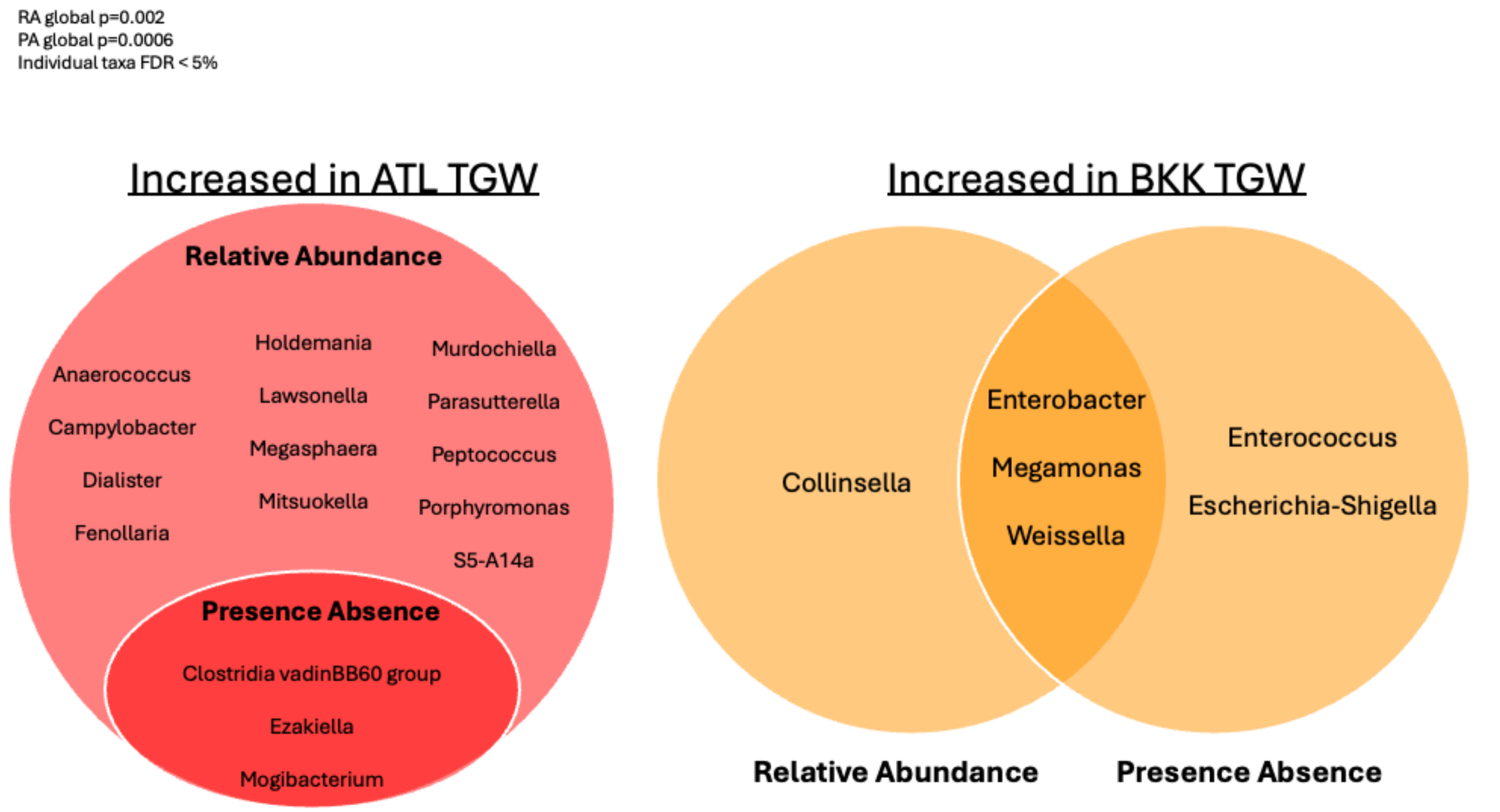

Supplemental Figure 4: Location Comparisons in Transgender Women by LDM. The rectal microbiota significantly differed between ATL TGW (transgender women in Atlanta; n=25) and BKK TGW (transgender women in Bangkok; n=97) by relative abundance linear decomposition modeling (LDM; global p=0.002; 20 differentially-abundant taxa) and presence-absence LDM (global p=0.0006; 8 taxa with differential probability of presence). Alpha diversity was not significantly different (p=0.052). Unidentified and uncultured taxa are not included in this figure.

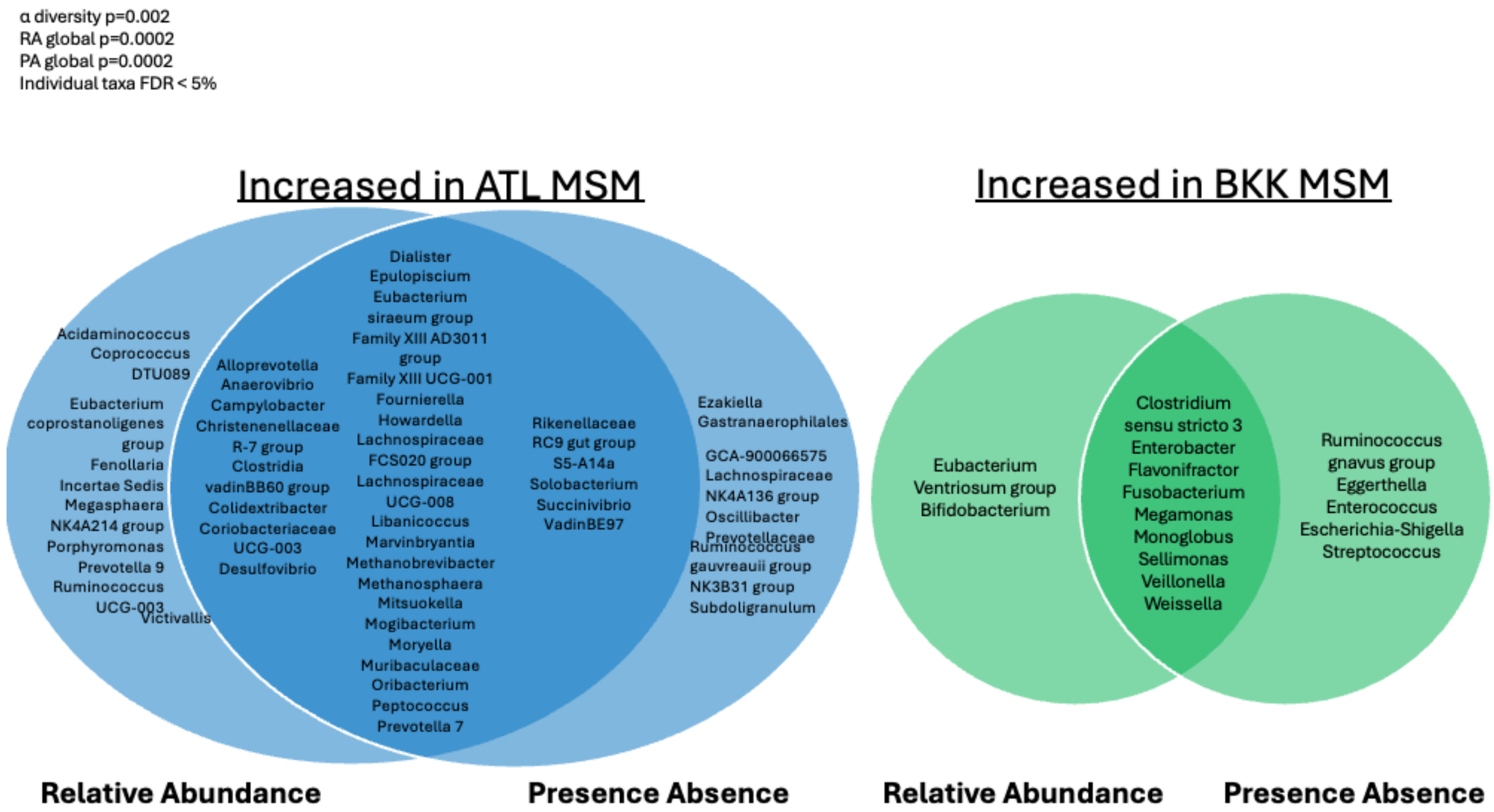

Supplemental Figure 5: Location Comparisons in Cisgender Men who Have Sex with Men by LDM. The rectal microbiota significantly differed between ATL MSM (cisgender men who have sex with men in Atlanta; n=33) and BKK MSM (cisgender men who have sex with men in Bangkok; n=50) by relative abundance linear decomposition modeling (LDM; global p=0.0002; 66 differentially-abundant taxa) and presence-absence LDM (global p=0.0006; 64 taxa with differential probability of presence). Alpha diversity was also significantly different (p=0.002). Unidentified and uncultured taxa are not included in this figure.

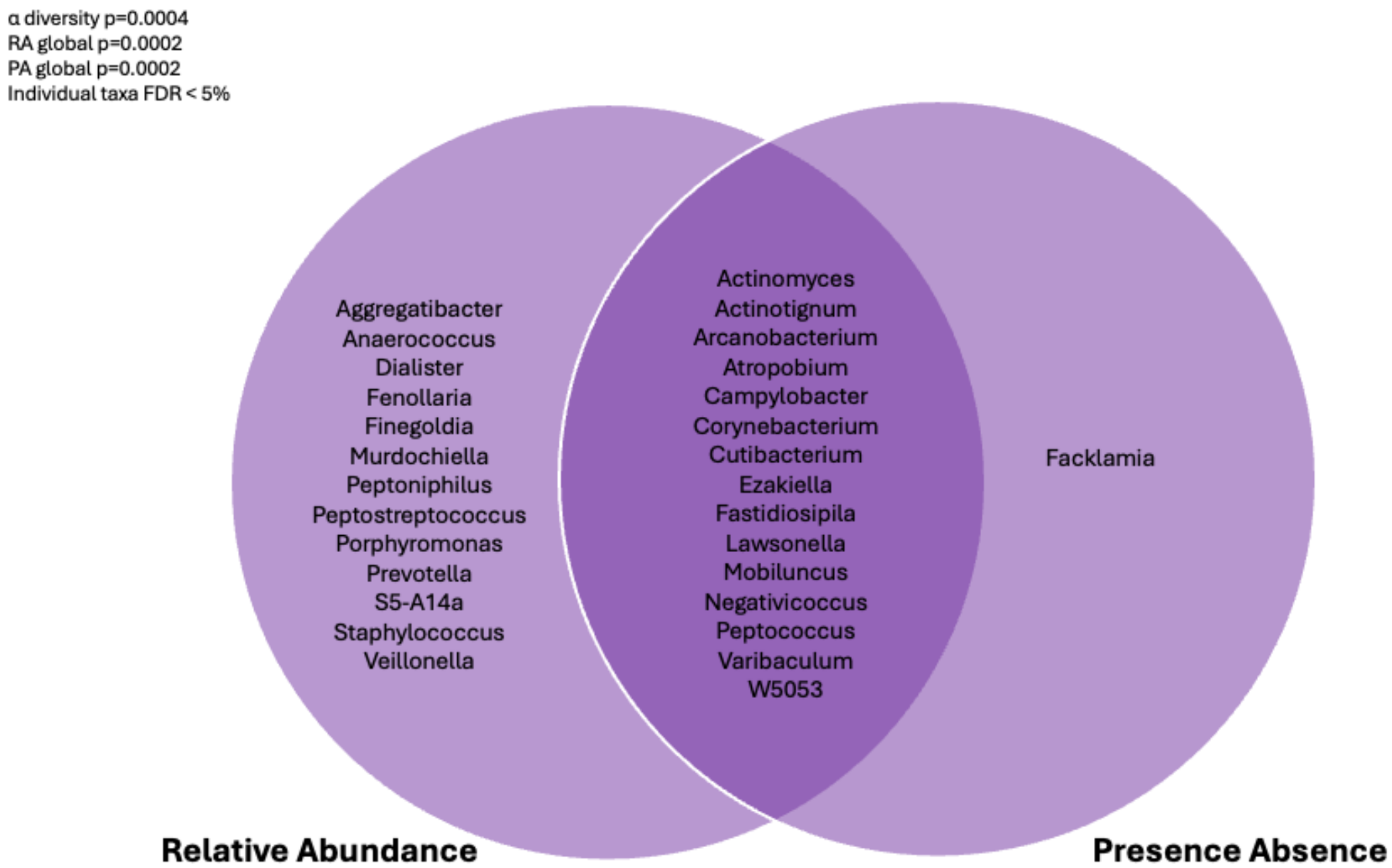

Supplemental Figure 6: Increased in Neovagina Compared to Rectum in Transgender Women in Bangkok. We compared the rectal (n=97) and neovaginal (n=13) microbiotas in a subset of transgender women in Bangkok. The microbiotas were globally significantly different by relative abundance linear decomposition modeling (LDM; global p=0.0002; 88 differentially-abundant taxa; 28 increased in the neovagina compared to the rectum) and presence-absence LDM (global p=0.0002; 86 taxa with differential probability of presence; 16 of these with increased probability of presence in the neovagina compared to the rectum). Alpha diversity was also significantly different (p=0.0004). Unidentified and uncultured taxa are not included in this figure.

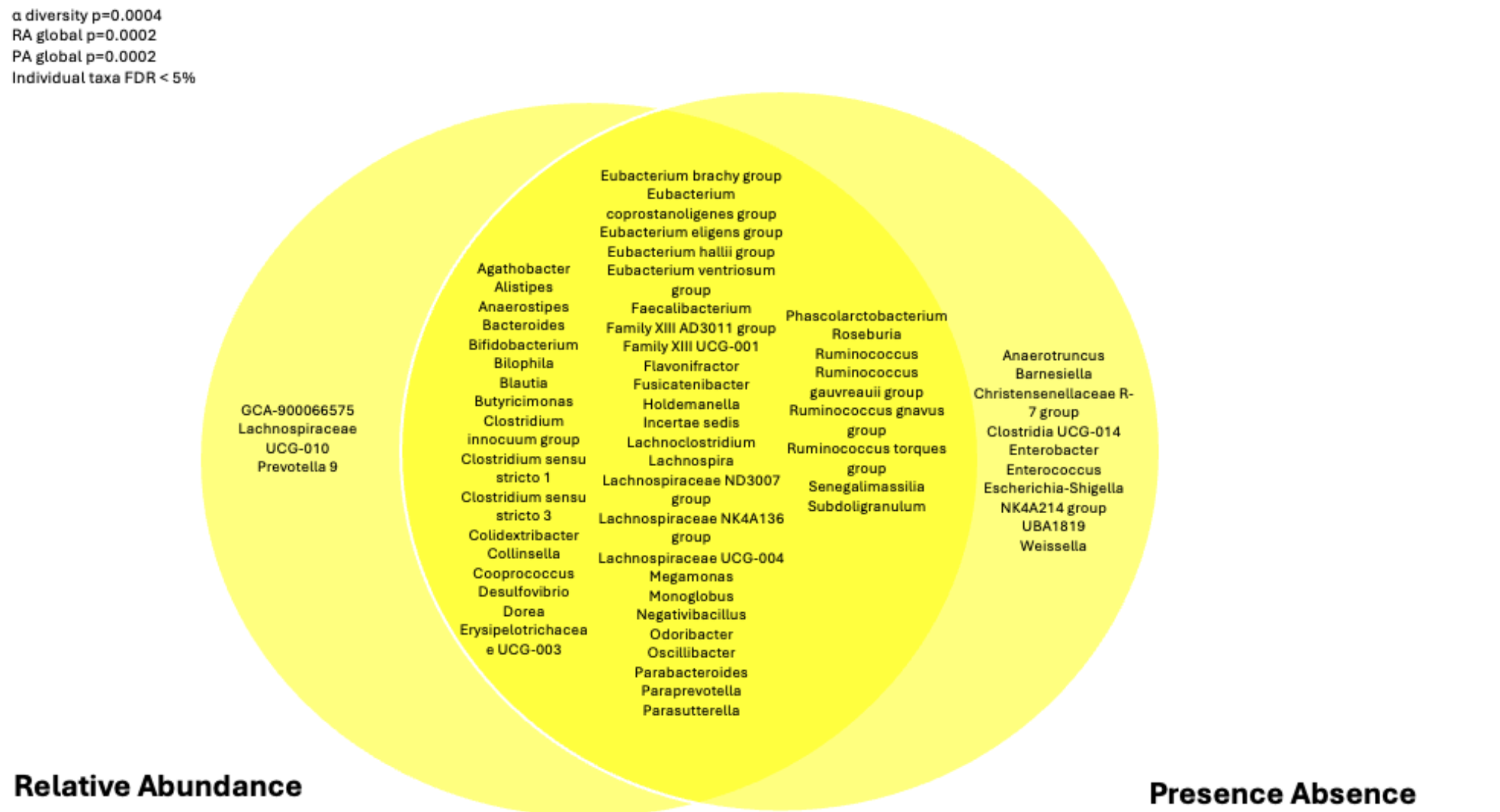

Supplemental Figure 7: Increased in Rectum Compared to Neovagina in Transgender Women in Bangkok. We compared the rectal (n=97) and neovaginal (n=13) microbiotas in a subset of transgender women in Bangkok. The microbiotas were globally significantly different by relative abundance linear decomposition modeling (LDM; global p=0.0002; 88 differentially-abundant taxa; 60 increased in the rectum compared to the neovagina) and presence-absence LDM (global p=0.0002; 86 taxa with differential probability of presence; 70 of these with increased probability of presence in the rectum compared to the neovagina). Alpha diversity was also significantly different (p=0.0004). Unidentified and uncultured taxa are not included in this figure.

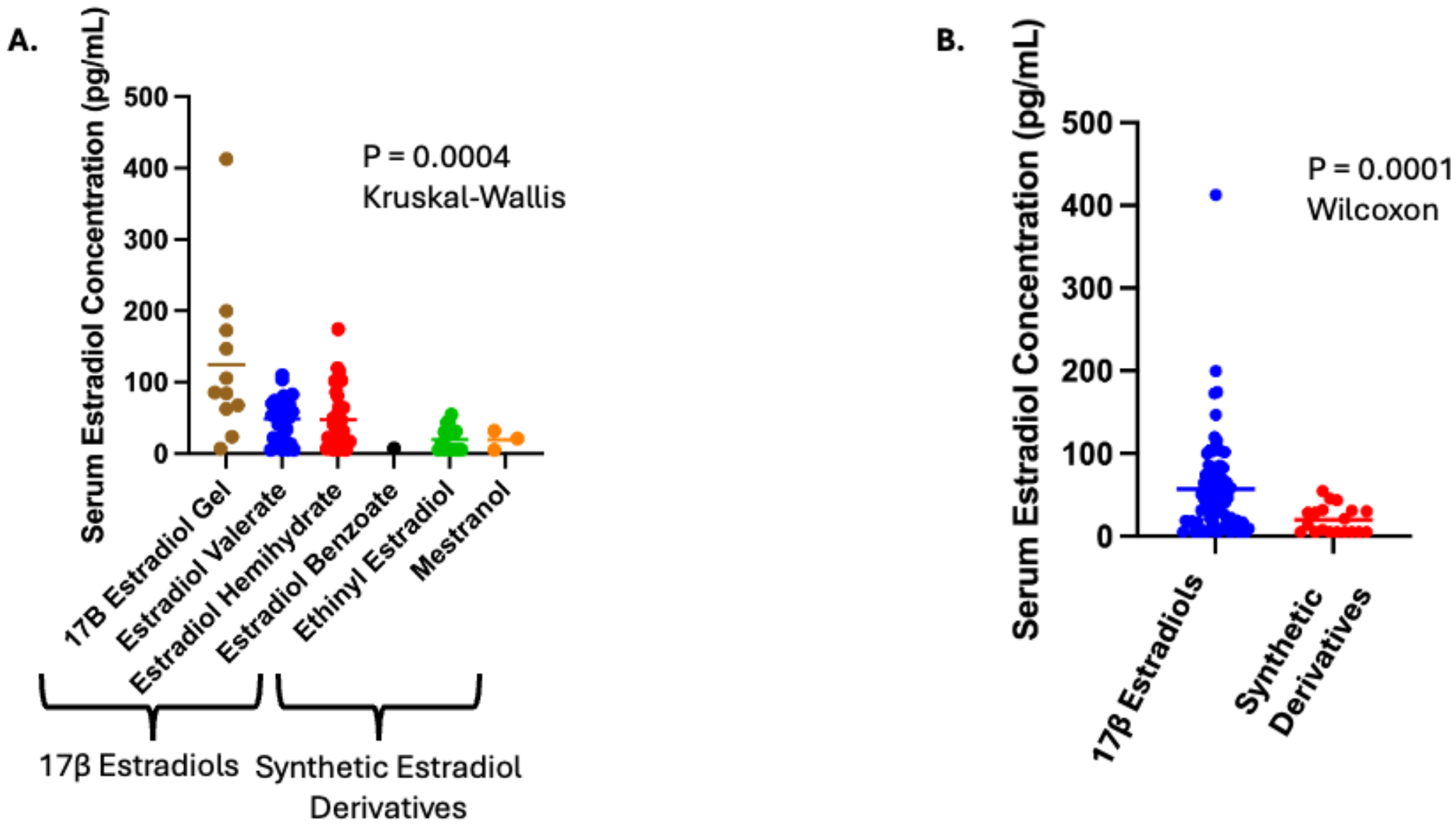

Supplemental Figure 8: Serum Estradiol Concentrations Stratified by Estradiol Formulation. (A) Depicts median serum estradiol concentrations for all TGW participants grouped by estradiol formulation. (B) Depicts median serum estradiol concentrations for all TGW participants grouped by bioidentical drug and prodrug versus synthetic estradiol derivative formulations.

Supplemental Tables

Taxa	R	P Value
Bacteroidaceae	0.15	0.03
Campylobacteraceae	0.21	0.003
Enterobacteriaceae	-0.14	0.04
Pasteurellaceae	-0.19	0.008
Porphyromonadaceae	0.23	0.001
Selenomonadaceae	-0.25	0.0003

Supplemental Table 1: Serum Estradiol and Family Level Microbiome Relative Abundance - Significant Spearman Correlations. These six phylogenetic families (of 39 total that were assessed) demonstrated significant ($p<0.05$) correlations between relative abundance and serum estradiol concentrations.

Taxa	R	P Value
Bacteroidaceae	-0.16	0.02
Lactobacillaceae	-0.2	0.005
Monoglobaceae	-0.2	0.007
Porphyromonadaceae	-0.15	0.03
Rikenellaceae	-0.2	0.004

Supplemental Table 2: Serum Testosterone and Microbiome Relative Abundance - Significant Spearman Correlations. These five phylogenetic families (of 39 total that were assessed) demonstrated significant ($p<0.05$) correlations between relative abundance and serum testosterone concentrations.
